# Predicting the response of a long‐distance migrant to changing environmental conditions in winter

**DOI:** 10.1002/ece3.11619

**Published:** 2024-06-29

**Authors:** R. A. Stillman, E. M. Rivers, W. Gilkerson, K. A. Wood, P. Clausen, C. Deane, D. H. Ward

**Affiliations:** ^1^ Department of Life and Environmental Sciences, Faculty of Science and Technology Bournemouth University Poole UK; ^2^ Merkel & Associates, Inc. San Diego California USA; ^3^ Wildfowl & Wetlands Trust Slimbridge, Gloucestershire UK; ^4^ Department of Ecoscience ‐ Wildlife Ecology Aarhus University Aarhus C Denmark; ^5^ Department of Biology and Wildlife University of Alaska Fairbanks Fairbanks Alaska USA; ^6^ U.S. Geological Survey, Alaska Science Center 4210 University Drive Anchorage Alaska USA

**Keywords:** Black Brant (*Brant bernicla nigricans*), eelgrass (*Zostera marina*), environmental change, food competition, individual‐based model

## Abstract

Access to high‐quality food is critical for long‐distance migrants to provide energy for migration and arrival at breeding grounds in good condition. We studied effects of changing abundance and availability of a marine food, common eelgrass (*Zostera marina* L.), on an arctic‐breeding, migratory goose, black brant (*Brant bernicla nigricans* Lawrence 1846), at a key non‐breeding site, Bahía San Quintín, Mexico. Eelgrass, the primary food of brant, is consumed when exposed by the tide or within reach from the water's surface. Using an individual‐based model, we predicted effects of observed changes (1991–2013) in parameters influencing food abundance and availability: eelgrass biomass (abundance), eelgrass shoot length (availability, as longer shoots more within reach), brant population size (availability, as competition greater with more birds), and sea level (availability, as less food within reach when sea level higher). The model predicted that the ability to gain enough energy to migrate was most strongly influenced by eelgrass biomass (threshold January biomass for migration = 60 g m^−2^ dry mass). Conversely, annual variation in population size (except for 1998), was relatively low, and variation in eelgrass shoot length and sea level were not strongly related to ability to migrate. We used observed data on brant body mass at Bahía San Quintín and annual survival to test for effects of eelgrass biomass in the real system. The lowest observed values of body mass and survival were in years when biomass was below 60 g m^−2^, although in some years of low biomass body mass and/or survival was higher. This suggests that the real birds may have some capacity to compensate to meet their energy demands when eelgrass biomass is low. We discuss consequences for brant population trends and conservation.

## Graphical abstract

We studied effects of annual changes in abundance and availability of a marine food, common eelgrass (*Zostera marina*), on an arctic‐breeding, migratory goose, black brant (*Brant bernicla nigricans*), at a key non‐breeding site, Bahía San Quintín, Mexico. Using an individual‐based model, we predicted that the ability to gain enough energy to migrate was most strongly influenced by eelgrass biomass, rather than eelgrass shoot length, brant population size or sea level. We compare these predictions to observations from the real system and discuss consequences for brant population trends and conservation.

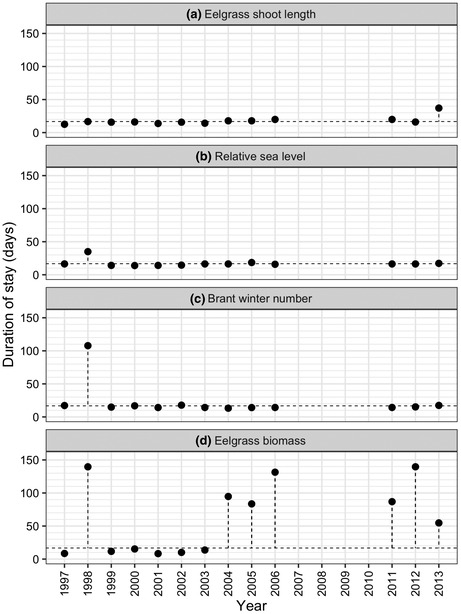

## 1 INTRODUCTION

Understanding how and when environmental conditions limit winter food supply is important for management and conservation of migratory bird populations because nutrients, and in turn energy reserves, acquired in winter may influence survival and fecundity (Kéry et al., 2006; Marra et al., 1998). Effects of reduced nutrients and energy on winter condition of migrants can be immediate leading to shifts in distribution (Lindberg et al., 2007) and increased mortality (Kirby et al., 1986; Inger et al., 2008). Winter food limitations also increase competition and may negatively and disproportionally affect first‐year birds because these migrants typically have lower overwinter and annual survival than adults (Francis et al., 1992; Leach et al., 2017; Pilotte et al., 2014; Schmutz et al., 1994; Ward et al., 2004). If effects are non‐lethal, the condition of birds in winter can carry‐over to other seasons and delay the timing of spring migration (Bêty et al., 2003) and reduce reproductive performance (Marra et al., 1998; Schamber et al., 2012; Sedinger et al., 2006). In essence, long‐distance migrants that have access to high‐quality foods in winter have a greater probability of breeding (Sedinger et al., 2011) and produce more offspring than those that have access to low‐quality foods in winter (Schamber et al., 2012; Sillett et al., 2000). Thus, food availability and abundance in winter, through its effect on energy reserves, can be a key driver of population dynamics for long‐distance avian migrants.

**FIGURE 1:**
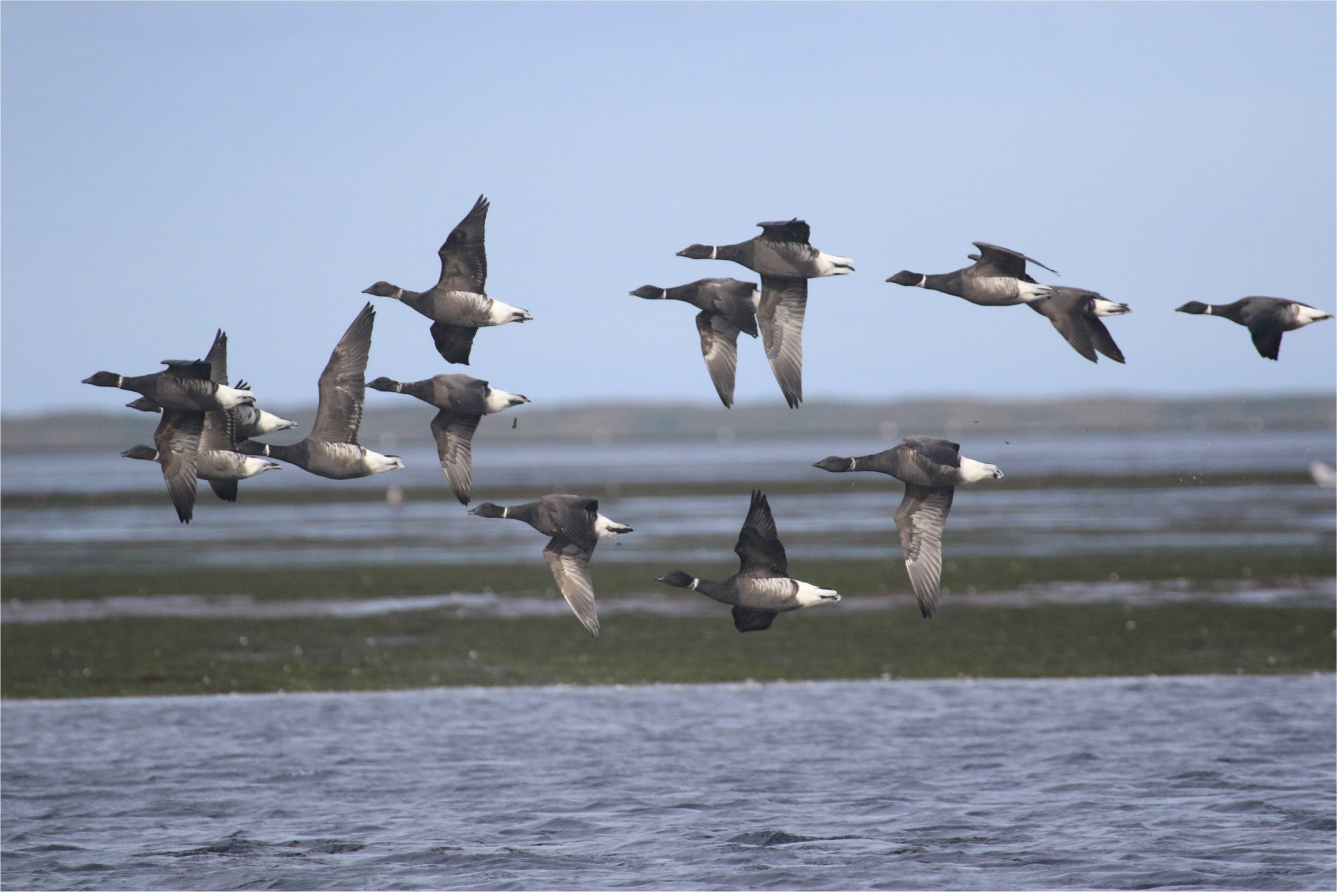
Flock of black brant (*Brant bernicla nigricans* Lawrence 1846) (termed, brant, in the text) with seagrass *Zostera marina* L. bed (termed, eelgrass, in the text) in the distance. Photograph credit: Maynard Axelson.

The eastern Pacific population of black brant (*Brant bernicla nigricans* Lawrence 1846) (hereafter, brant) (Figure 1) wintering in Mexico is declining, where these arctic‐breeding birds have traditionally spent their nonbreeding season (November–May; Lewis et al., 2020). Numbers of brant in Mexico have dropped nearly 33% between 2000 and 2022 despite an overall population that has remained stable or slightly declined over the same period (Olson, 2022; Sedinger et al., 2019). The decline of brant may be driven by decreasing abundance of their primary winter food (Ward et al., 2003, 2005). Brant rely solely on intertidal habitats and feed almost exclusively on the seagrass *Zostera marina* L. (hereafter, eelgrass) during the nonbreeding season (Lewis et al., 2020; Ward et al., 2005). Climate warming and its associated components, such as increasing sea level, sea surface temperatures, and intensity of storms are altering seagrasses and other coastal marine habitats (Lefcheck et al., 2017; Orth et al., 2006; Ward et al., 2003; Waycott et al., 2009). These factors pose a threat to eelgrass populations in northwest Mexico (Shaughnessy et al., 2012; Ward et al., 2003) because this region represents the southern limit of the distributional range of eelgrass in the northeastern Pacific (Wyllie‐Echeverria & Ackerman, 2003) and eelgrass populations are hence already under maximal thermal stress from irradiance and desiccation (Cabello‐Pasini et al., 2003; Meling‐López & Ibarra‐Obando, 1999). Short‐term increases in sea temperature and sea level, associated with El Niño Southern Oscillation (ENSO) events, cause reduction in eelgrass production and biomass (Cabello‐Pasini et al., 2002; Echavarria‐Heras et al., 2006; Johnson et al., 2003; Thom et al., 2003), reducing the amount of food available to brant. Furthermore, short‐term increases in sea level, mean that on average eelgrass will be covered by a greater depth of water/exposed by the tide for less time, reducing the availability of eelgrass to brant, which are limited to foraging to depths of approximately 0.4 m (Clausen, 2000).

In this study, we use a combination of observed data and an individual‐based model (IBM, called MORPH; Stillman, 2008) to examine potential threats to brant at a primary wintering and spring staging area, Bahía San Quintín, Mexico, by predicting how changes in environmental conditions are likely to affect their ability to migrate northward to the breeding grounds. IBMs are an important tool for predicting individual or group interactions with their food supply and have been used extensively to evaluate the responses of foraging animals to changes in their environment (e.g., Brown & Stillman, 2021; Stillman, 2008; Stillman et al., 2015; Stillman & Goss‐Custard, 2010; Wood et al., 2012). We initially use observed data from 1991 to 2020 to highlight the importance of Bahía San Quintín as a wintering site in Mexico and to determine annual variation in biotic (eelgrass biomass and shoot length, brant population size) and abiotic (relative sea level) factors at the site that could potentially affect the ability of brant to migrate. We then use an IBM to predict for 13 years between 1997 and 2013 (years in which all variables were measured) the environmental conditions (i.e., combinations of eelgrass biomass and shoot length, brant population size and relative sea level) under which brant could have more difficulty gaining enough mass to survive and migrate from the site. The IBM is based on previous models of brant at other sites in the Pacific Flyway, Humboldt Bay, California (Stillman et al., 2015) and Izembek Lagoon, Alaska (Stillman et al., 2021). Finally, we test whether the observed brant body mass at Bahía San Quintín and annual survival varied between the years/environmental conditions in which the model either did or did not predict that brant would have difficulty surviving and migrating. Results from this study can better guide management, policymaking, and conservation decisions for this threatened brant population in Mexico (Danemann, 2018; Perez‐Arteaga & Gaston, 2004).

## 2 METHODS

### 2.1 Study system

**FIGURE 2:**
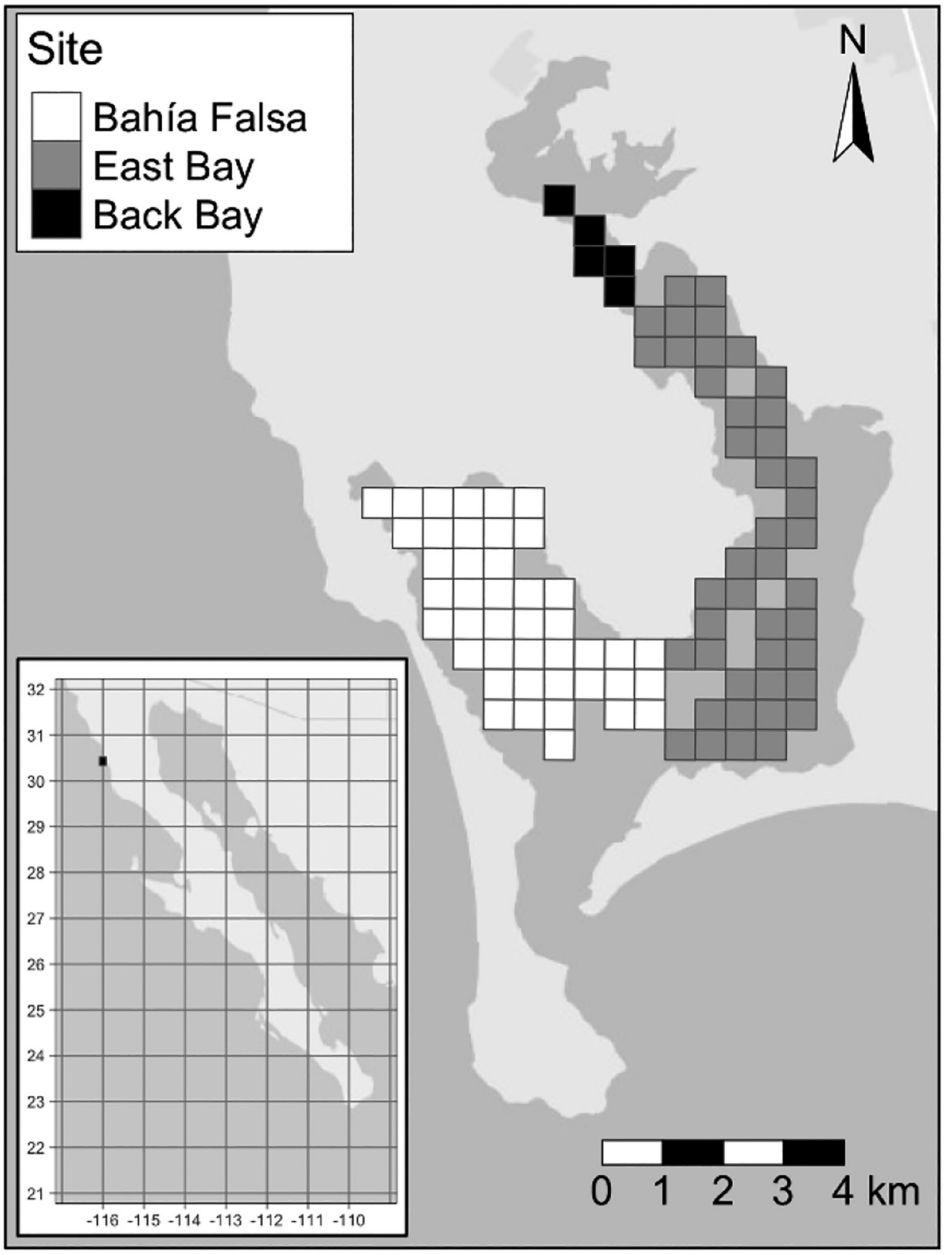
Map of Bahía San Quintín and its location within Mexico, showing the distribution of eelgrass patches included in the model. The symbol shading identifies patches within subsites of the bay. Latitude and longitude are shown on the insert map.

Bahía San Quintín is a 48 km^2^, shallow water coastal embayment in northwest Baja California, Mexico (30°25′ N, 115°58′ W) (Figure 2) and the most northerly primary wintering site for brant in Mexico. The bay annually supports a majority of brant that winter in Mexico, serving as the primary staging and stopover site during southward and northward migration, and a main wintering destination for the Mexican population (ca. 55% of birds either migrating through or overwintering in the bay) (Lewis et al., 2020; Lindberg et al., 2007; Palacios & Heredia, 2021). Bahía San Quintín is also a major sport hunting site of brant during winter (Kramer et al., 1979). Based on band recoveries the wintering population in Mexico is comprised of birds from a mixture of breeding areas, but largest percentage of breeders originates from the Yukon‐Kuskokwim Delta (YKD), Alaska (Leach et al., 2018), where most brant have traditionally nested (Sedinger et al., 1993). Eelgrass is the dominant marine macrophyte and the primary food of brant in the bay (Ward, 2022a, 2022b; Ward et al., 2003). Brant do not dive, but they feed on exposed intertidal or shallow subtidal eelgrass during low tides, which occur twice daily. Eelgrass is abundant in Bahía San Quintín, with biomass two to three times greater in this bay than in other primary wintering sites for brant in Mexico (Ward, 2022b). Other marine macrophytes, such as *Ruppia maritima* and *Ulva* spp., occur at much lower densities (Ibarra‐Obando & Aguilar‐Rosas, 1985; Ward, 2022b; Ward et al., 2003), but can provide food for brant when eelgrass biomass is low. There are no terrestrial food resources available for brant as the surrounding landmass in northwest Mexico is arid.

### 2.2 Observed annual variation in brant number, sea level, and eelgrass biomass and shoot length

We used the mid‐winter population index to assess annual trends in the number of the brant wintering in Mexico and Bahía San Quintín 1990–2020. Brant are surveyed annually during mid‐January by ground and aerial ocular counts at major use areas along west coasts of the Baja California Peninsula, Sonora and Sinaloa (Olson, 2022; Palacios & Heredia, 2021). The survey occurs over several days and is timed to occur when brant have reached their primary wintering destination.

Hourly changes in tides and interannual variation in sea level (m) were determined from tide gauge measurements made in San Diego Bay (https://www.tidesandcurrents.noaa.gov/), the nearest site (250 km away) with a continuous record of these measurements. We found that tide changes were similar between Bahía San Quintín and San Diego Bay (tide gage station: Broadway) for a subset of data from both bays (see Appendix S1 for details); therefore, we assumed that the tide and sea level estimates for San Diego Bay were also representative of relative changes in these parameters in Bahía San Quintín. Annual trends in relative sea level were determined from the average of mean monthly sea level estimates between November and April 1990–2020.

Eelgrass aboveground biomass (g m^−2^) and shoot length (m, meristem to longest leaf) were sampled at low tide during January (except 2 of 8 sites sampled in 2012 were made in December) at fixed sites (1997–2006 and 2011–2013) and along 50‐m‐long transects (2012–2013) placed in the mid to high (−0.2 to 0.5 m mean lower low water (mllw)) and low (−0.7 to −0.3 m mllw) intertidal of major eelgrass beds in Bahía San Quintín (Ward, 2022a). At each site or transect 4–6, 0.1 or 0.25 m^−2^ quadrats were randomly placed and all eelgrass shoots within each quadrat clipped. Samples were cleaned, and all dead material, rhizomes and flowering shoots were removed. Representative shoots were measured for length and all shoots dried to constant mass and weighed to determine aboveground biomass. The estimates for each quadrat were then scaled to g m^−2^ and averaged across all samples to calculate an annual biomass estimate.

We used linear models to test the relationships between year and the following six variables: (i) total mid‐winter brant numbers counted in Mexico, (ii) mid‐winter brant numbers counted at Bahía San Quintín, (iii) the proportion of brant mid‐winter numbers in Mexico that were present at Bahía San Quintín, (iv) sea level, (v) eelgrass biomass, and (vi) eelgrass shoot length. One of the key assumptions of such regression‐based analyses is the independence of model residuals, yet certain types of ecological data may violate that assumption. For example, count data for long‐lived waterfowl species may be more similar in adjacent years compared to distant years, which can lead to temporal autocorrelation in model residuals (Wood et al., 2019). We therefore tested for temporal autocorrelation in each of our six regression models by fitting generalized least squares models using the nlme package (Pinheiro et al., 2023) and examined for statistically significant temporal autocorrelation at each time lag in each regression model. We adjusted the *p* values to account for the multiple tests that we were carrying out across our six regression analyses, based on the methodology of Holm (1979). These analyses were conducted in R version 4.2.2 (R Core Team, 2022). We detected statistically significant temporal autocorrelation at time lag 1 for the sea level regression model, and so for the sea level data we used a first‐order moving average model (Crawley, 2013) for subsequent inference. No statistically significant temporal autocorrelation at any time lag was detected for any of the other temporal regressions, and therefore no autocorrelation structures were included in these models.

### 2.3 Predicted effects on brant of sea level, brant number, eelgrass biomass, and shoot length

The IBM used to predict the effect on brant of annual changes in conditions was based on previous models developed for brant at two sites in the USA: Humboldt Bay (Stillman et al., 2015) and Izembek Lagoon (Stillman et al., 2021). This section gives an overview of the model but full details of the model, its components and sources for parameter values are given in Appendix S1. Tests of the model, comparing its predictions to observations from the real system, are given in Appendix S2.

Model simulations ran in 1‐h time steps from 1 August to 15 May. The model defined periods of brant usage in the bay as “fall,” 1 August to 15 December; “winter,” 16 December to 15 February; and “spring,” 16 February to 15 May. These seasonal dates were based on changes in brant numbers during biweekly ground surveys within Bahía San Quintín over the study years and timing of use by radio‐tagged birds in one of the years (Ward, 2024). The model incorporated the diurnal (day or night), lunar (proportion of full moon), and tidal cycles (tidal height), with each time step occurring during the day or night, night‐time time steps potentially being moonlit, and water level changing between time steps.

The model included three subsites, termed Bahía Falsa, East Bay, and Back Bay (Figure 2). Across all subsites, the model divided space into 91, 500 × 500 m patches, each with a fixed shore elevation. The water depth over each patch during each time step was calculated as the difference between tidal height during the time step, and the elevation of the patch (see Appendix S1 for details).

One potential food resource was included in the model, eelgrass, distributed across the 500 × 500 m patches. Brant could potentially feed on eelgrass rooted within a patch (termed rooted eelgrass), or eelgrass that had become detached and was floating within a patch (termed floating eelgrass). The rooted eelgrass biomass and shoot length at the start of simulations were derived from bay‐wide surveys (see Appendix S1 for details). Eelgrass had a seasonally changing biomass (g m^−2^) and shoot length (m), and its biomass could also be reduced due to consumption by brant. Details of the seasonal changes in eelgrass biomass and shoot length are given in Appendix S1. The biomass of floating eelgrass within each patch was also incorporated into the model as a fixed proportion (0.05) of the biomass of rooted eelgrass growing in the patch (e.g., if the rooted biomass was 10 g m^−2^, the floating biomass would be 0.5 g m^−2^). Brant were able to feed on rooted eelgrass if it was exposed by the tide or within reach from the water surface (determined by tidal height, shore elevation of patch, and eelgrass shoot length) and could feed on floating eelgrass at any stage of the tide. Eelgrass had a specific energy content (KJ g^−1^) and digestibility (proportion of energy in food assimilated) which determined its food value for the birds (see Appendix S1 for details). Rooted and floating eelgrass were the only food resources included within the model.

The model included three types of brant: fall migrants—birds that passed through the site during southward migration; spring migrants—birds that passed though the site during northward migration; and winter residents—birds that spent the winter in the site. Because of the large number of brant that used the site, model simulations used flocks comprised of 100 individuals (i.e., “super‐individuals” sensu Scheffer et al., 1995) rather than simulating each individual goose. This meant that each model flock (individual) was equivalent to 100 real individuals. Each model flock was randomly assigned a date when it arrived, drawn from a uniform distribution between the observed first and last arriving brant of each type (see Appendix S1 for details).

The model tracked the amount of energy stored by each brant, calculated as body mass minus lean body mass, and multiplied by the energy content of fat (see Appendix S1 for details). Brant were also assigned a body mass on arrival in the system (see Appendix S1 for details). Fall migrant brant were assumed to emigrate from the site a fixed number of days after their arrival, irrespective of their body mass, whereas over‐winterers and spring migrant brant were assumed to remain in the system until a specific departure day and departure energy store were reached (see Appendix S1 for details).

When present in the site, each model brant had a seasonally dependent energy requirement (KJ hr^−1^) and seasonally dependent target energy store size (KJ) which it attempted to meet by feeding on eelgrass (see Appendix S1 for details). Following Stillman et al. (2021), the rate at which the model brant could consume eelgrass was calculated using a functional response, relating the biomass of food to the rate of consuming food (see Appendix S1 for details). The model assumed that the rate of feeding (for a fixed biomass) was the same during daylight and moonlit nights and brant fed on either rooted or floating eelgrass, depending on which resource type maximized their rate of energy assimilation (see Appendix S1 for details). The model incorporated competition between the birds due to resource depletion, with the food available to birds within a patch during a time step depending on the previous depletion through consumption by the birds plus seasonal changes in biomass due to other factors (see Appendix S1 for details).

During each time step, the model predicted which patch each flock occupied based on the tidal availability of food and the rate at which energy could be assimilated from the food consumed (see Appendix S1 for details). Model birds moved to the patch and consumed the resource which maximized their rate of assimilating energy (KJ hr^−1^). If birds were able to assimilate energy at a high enough rate, they were able to meet their energy requirements and maintain, or increase, their target energy store size. If birds were not able to meet their energy requirements, they needed to draw on their energy store and so the overall size of their energy store was reduced. Fall migrants departed from the site after they had been present for 11 days, the mean length of stay in the site for migrants wintering south of this bay (Ward, 2024). Brant migrated from the site during winter or spring if their energy stores were large enough after a specific date (see Appendix S1 for details). If the size of a bird's energy store fell to zero it died of starvation.

Two types of simulations were run to predict the effects on brant of variation in four parameters, relative sea level (ENSO cycles), overwintering brant population size, eelgrass biomass, and shoot length. The first set of simulations termed the *baseline simulations*, predicted the effect on brant of annual variation in observed data for relative sea level and brant overwinter population size between 1991 and 2020, and eelgrass biomass and shoot length for 13 years, 1997–2006 and 2011–2013 (see Appendix S1 for parameter values used for each year). Simulations for each year did not include any carryover effects from previous years, with the parameter values at the start of 1 year not dependent on any predictions made for the previous year. The second set of simulations, termed the *single variable simulations*, were run to determine which of the four parameters had the greatest impact on the duration of stay for spring migrant brant. Predictions were restricted to spring migrants as they were expected to be most sensitive to changes in parameter values. In these simulations, one parameter was varied between years with the other three parameters held at their mean value across years. The importance of each parameter was assessed from the difference between the predicted values when the parameter varied between years compared to the predicted value when all parameters were held at their mean value.

The model incorporated stochasticity in terms of the arrival dates of birds. Five replicate simulations were run for the baseline simulations, with mean predictions and associated 95% confidence intervals presented. These simulations showed that model predictions were consistent between replicates (see below); therefore, only one replicate was run in the single variable simulations.

The baseline model was tested by comparing as many of its predictions as possible to observations from the study site. Observations were made during different ranges of years over the study period. Therefore, observations were compared to predictions that were derived from a similar range of years. The results of model testing are given in Appendix S2.

### 2.4 Observed relationships between eelgrass biomass and brant body mass and survival

The model simulations predicted that the difficulty brant had in migrating was more strongly related to year‐to‐year variation in eelgrass biomass than to variation in sea level, brant number, or shoot length (see below for details). These simulations also predicted a threshold eelgrass biomass, below which birds were predicted to have difficulty in migrating (see below for details). To evaluate the influence of year‐to‐year variation in eelgrass biomass on the modelled birds, observed data on annual mean estimates of body mass of brant at Bahía San Quintín and annual survival of brant breeding on the YKD, Alaska were compared between years with an eelgrass biomass either above or below the threshold. Briefly, estimates of body mass of brant were collected from birds shot by sport hunters in January (see Ward, 2024). Annual survival estimates were based on thousands of resightings and recoveries of marked‐ birds made throughout the annual cycle (see Leach et al., 2017). Comparisons of body mass and survival were made within age class (first‐year/adult) and sex (male/female) combinations between years either above or below the threshold biomass.

## 3 RESULTS

### 3.1 Observed annual variation in brant number, sea level, and eelgrass biomass and shoot length

**FIGURE 3:**
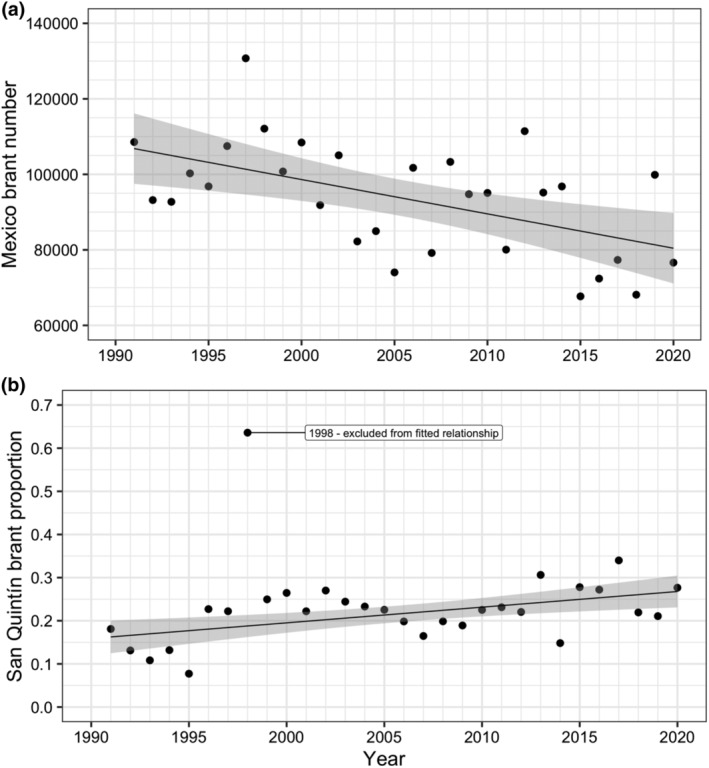
Observed relationships between year and (a) brant population size in Mexico during January and (b) the proportion of Mexico brant population in San Quintín during January 1991–2000. Lines show relationships fitted using linear regression (all years except for brant proportion in 1998) and grey shading indicate 95% confidence interval of fitted relationships.

The number of brant wintering in Mexico declined significantly between 1991 and 2020 (linear regression: brant number = 1,918,118–909.7 × Year; *n* = 30; *R* ^2^ = 0.289; *p* = .002; Figure 3a), with the proportion of the Mexico brant population wintering in Bahía San Quintín increasing significantly during this period (linear regression: proportion of brant = −7.0635 + 0.0036 × Year; *n* = 29 (1998 excluded); *R* ^2^ = 0.297; *p* = .002; Figure 3b). One exceptional year was 1998, during which the proportion of brant wintering in Bahía San Quintín, was considerably greater (approx. 0.6) than other years (approx. 0.1–0.35).

**FIGURE 4:**
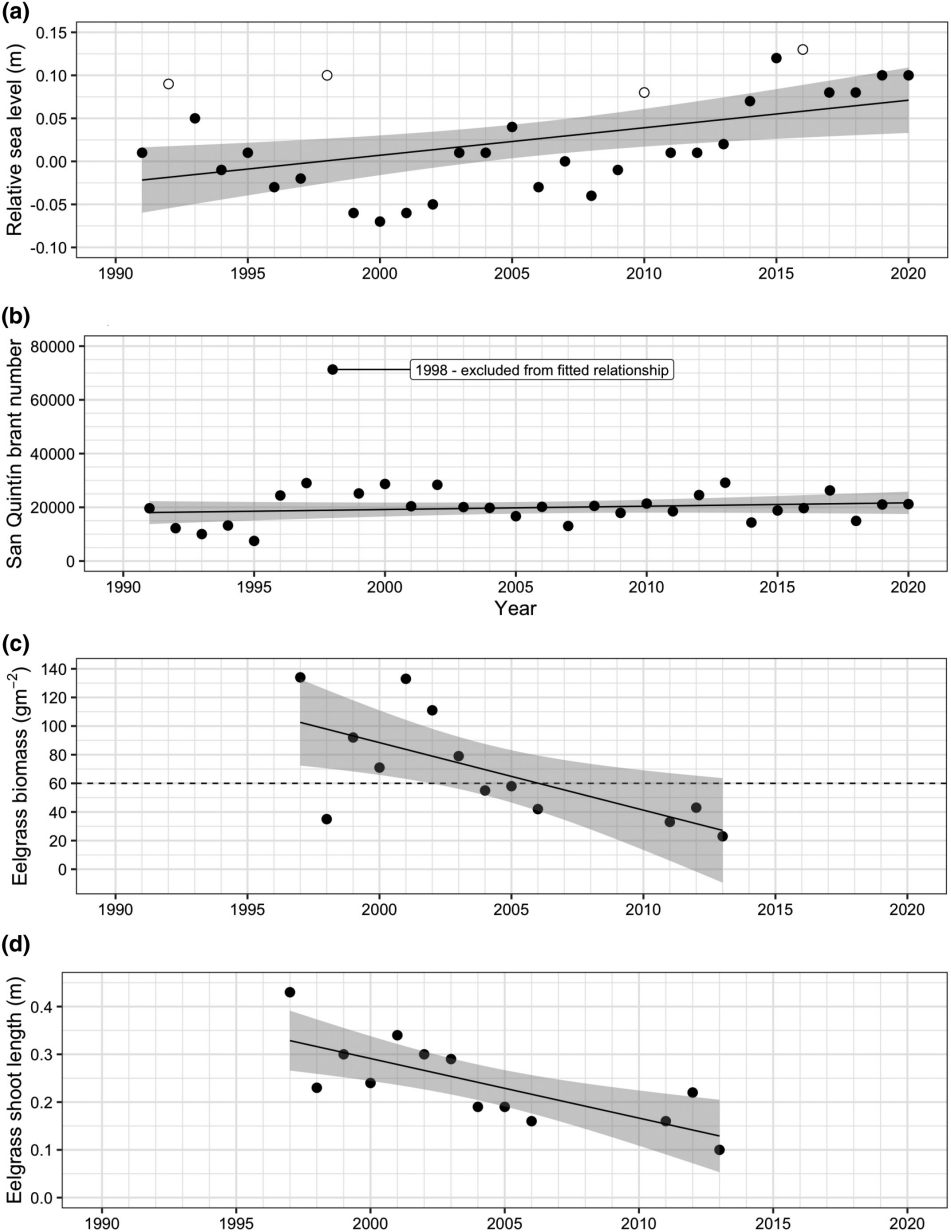
Observed relationships between year and (a) relative sea level (open symbols = years with extreme ENSO events (Multivariate ENSO Index (MEI) Index Scores >1.0)), (b) brant population size, (c) eelgrass biomass, and (d) eelgrass shoot length in Bahía San Quintín during January. Lines show relationships fitted using linear regression (all years except brant numbers in 1998) and gray shading indicate 95% confidence interval of fitted relationships. Horizontal dashed line in (c) indicates an eelgrass biomass of 60 g m^−2^.

Relative sea level increased significantly between 1991 and 2020 (first‐order autoregressive model: sea level (m) = −6.5240 + 0.0033 × Year; *n* = 30; *R* ^2^ = 0.232; *p* = .03) and varied considerably year‐to‐year, driven by the ENSO cycles (Figure 4a). Several years had exceptionally high sea levels, with 1998 being one of the most extreme (1998 relative sea level approx. 0.1 m, compared to fitted value of regression line approx. 0.0 m). The number of brant wintering in Bahía San Quintín did not change significantly through time (linear regression: brant number = −230,765 + 125.0 × Year; *n* = 29 (1998 excluded); *R* ^2^ = 0.038; *p* = .313) but was considerably greater in 1998 (approx. 70,000 birds) than in other years (10,000–30,000 birds; Figure 4b). Eelgrass biomass and shoot length declined significantly during this time (linear regression: eelgrass biomass (g m^−2^) = 9519–4.715 × Year; *n* = 13; *R* ^2^ = 0.443; *p* = .013; Figure 4c; eelgrass shoot length (m) = 25.24–0.0125 × Year; *n* = 13; *R* ^2^ = 0.563; *p* = .003; Figure 4d).

### 3.2 Predicted effects on brant of sea level, brant number, eelgrass biomass, and shoot length

Baseline simulations predicted that all fall migrant brant survived in all years, but that less than 100% of winter residents and spring migrants survived in 1998, 2011, and 2013. During these years, predicted survival of winter residents was zero, and predicted survival of spring migrants was 55%, 65%, and 38% in 1998, 2011, and 2013, respectively. Baseline simulations predicted that all fall migrant brant emigrated from the site in all years, but that no winter residents or spring migrants migrated by the end of the simulation (i.e., 15 May) during 1998, 2004, 2006, 2011, 2012, and 2013.

**FIGURE 5:**
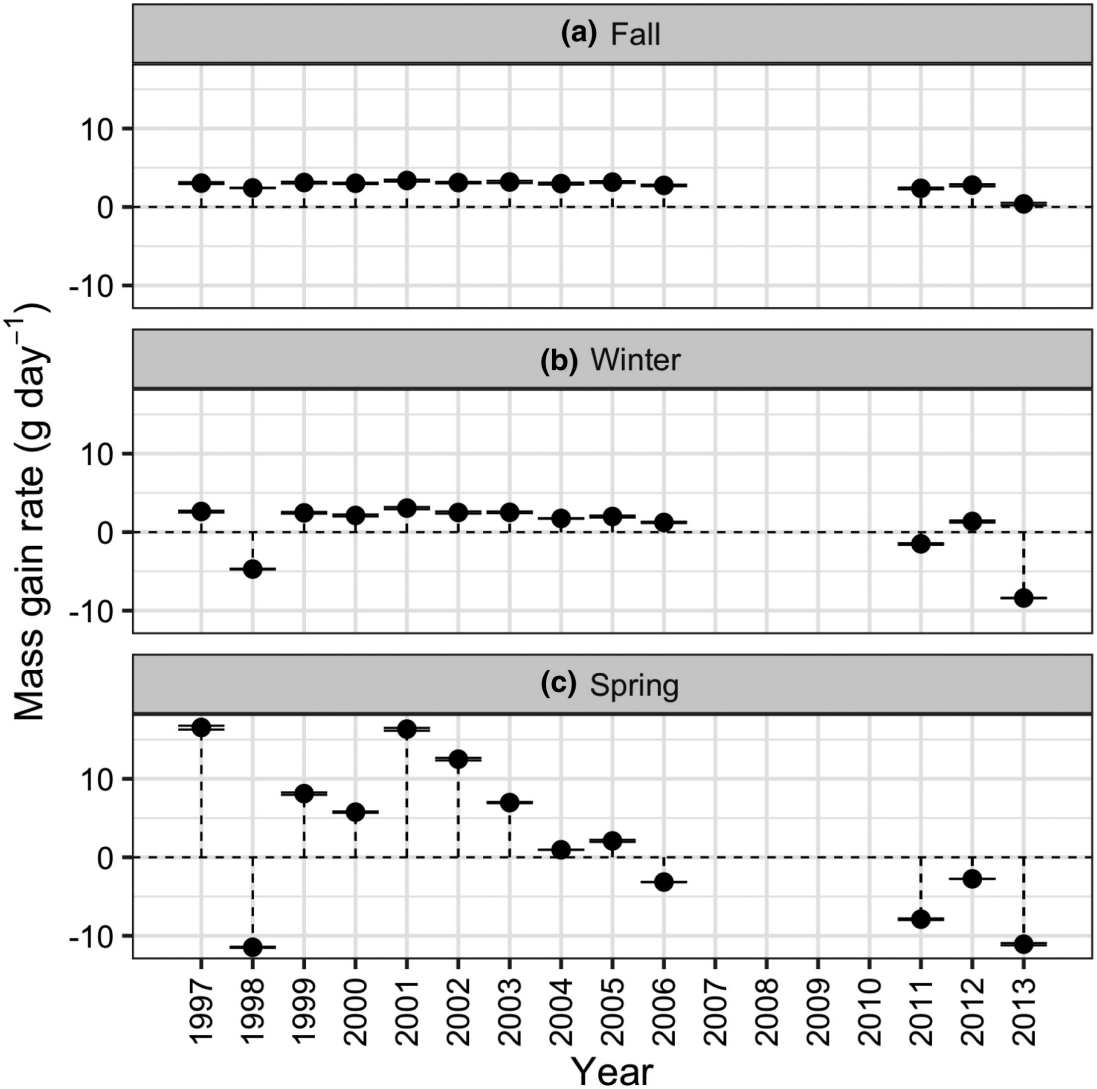
Predicted between‐year variation in the rate of mass gain of brant during (a) fall, (b) winter, and (c) spring. Relative sea level, eelgrass biomass and shoot length, and brant winter population size varied between years, with remaining parameters unchanged. Symbols show mean predictions of five replicate simulations and error bars indicate associated 95% confidence intervals (error bars appear as a single line when 95% confidence intervals are small). The broken horizontal line shows a mass gain of 0 g day^−1^; symbols above this line show mass gain and symbols below show mass loss.

**FIGURE 6:**
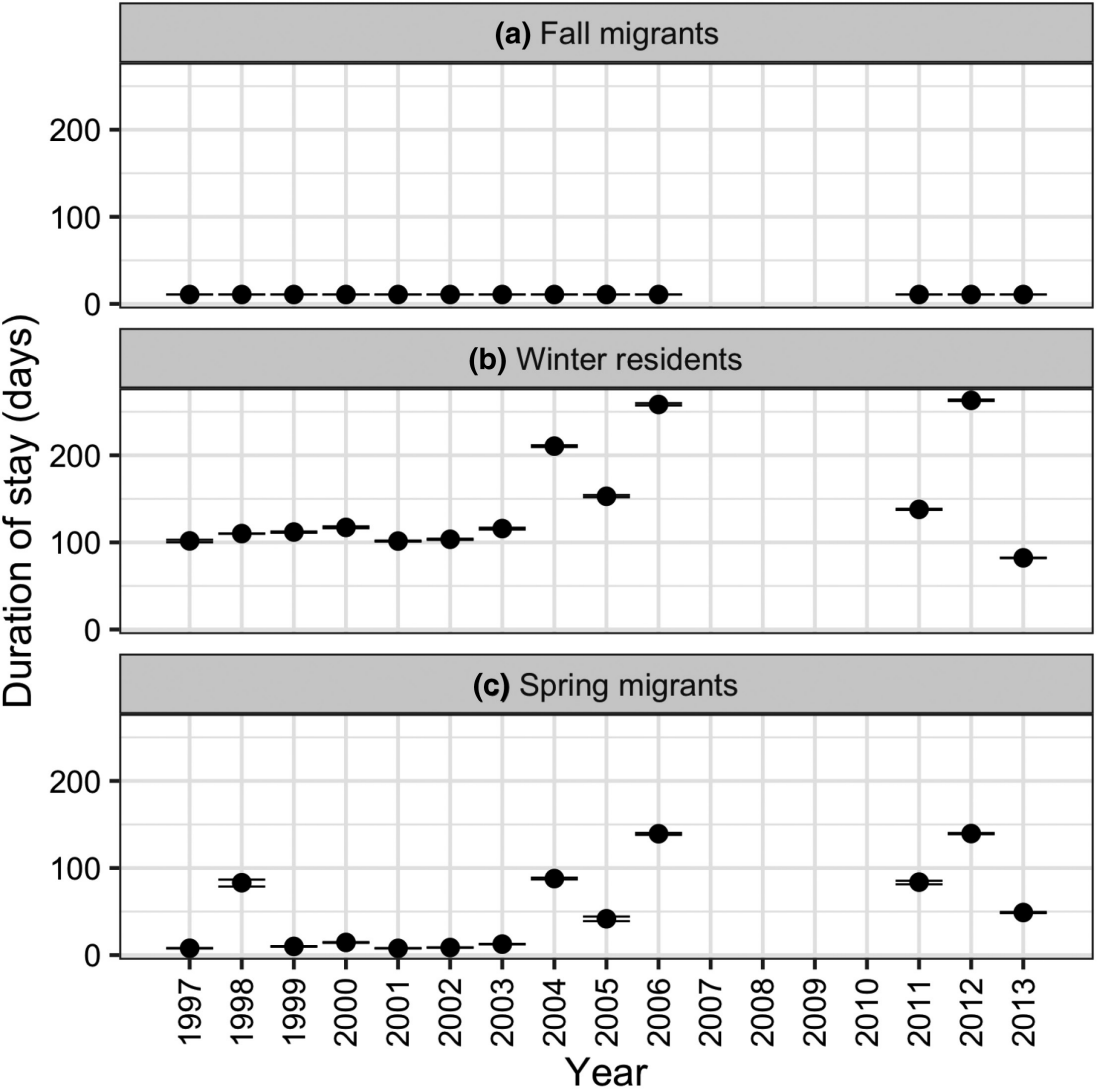
Predicted between‐year variation in the duration of stay of (a) fall migrants, (b) winter residents, and (c) spring migrants. Relative sea level, eelgrass biomass and shoot length, and brant winter population size varied between years, with remaining parameters unchanged. Symbols show mean predictions of five replicate simulations and error bars indicate associated 95% confidence intervals (error bars appear as a single line when 95% confidence intervals are small).

Baseline simulations showed that the predicted rate of mass gain (Figure 5a) and duration of stay of fall migrants (Figure 6a) did not vary greatly among years—the duration of stay in fall was not expected to vary as it was fixed to 11 days (see Appendix S1 for details) but is presented for completeness. In contrast, the predicted rate of mass gain (Figure 5b,c) and duration of stay for winter residents and spring migrants (Figure 6b,c) varied more between years. The greatest year‐to‐year variation occurred for predicted rate of mass gain and duration of stay of spring migrants, with rate of mass gain lowest and duration of stay greatest in 1998, and 2004–2013. Therefore, the baseline model predicted that brant had more difficulty meeting their energy requirements and migrating from the site during 7 of the 13 study years.

**FIGURE 7:**
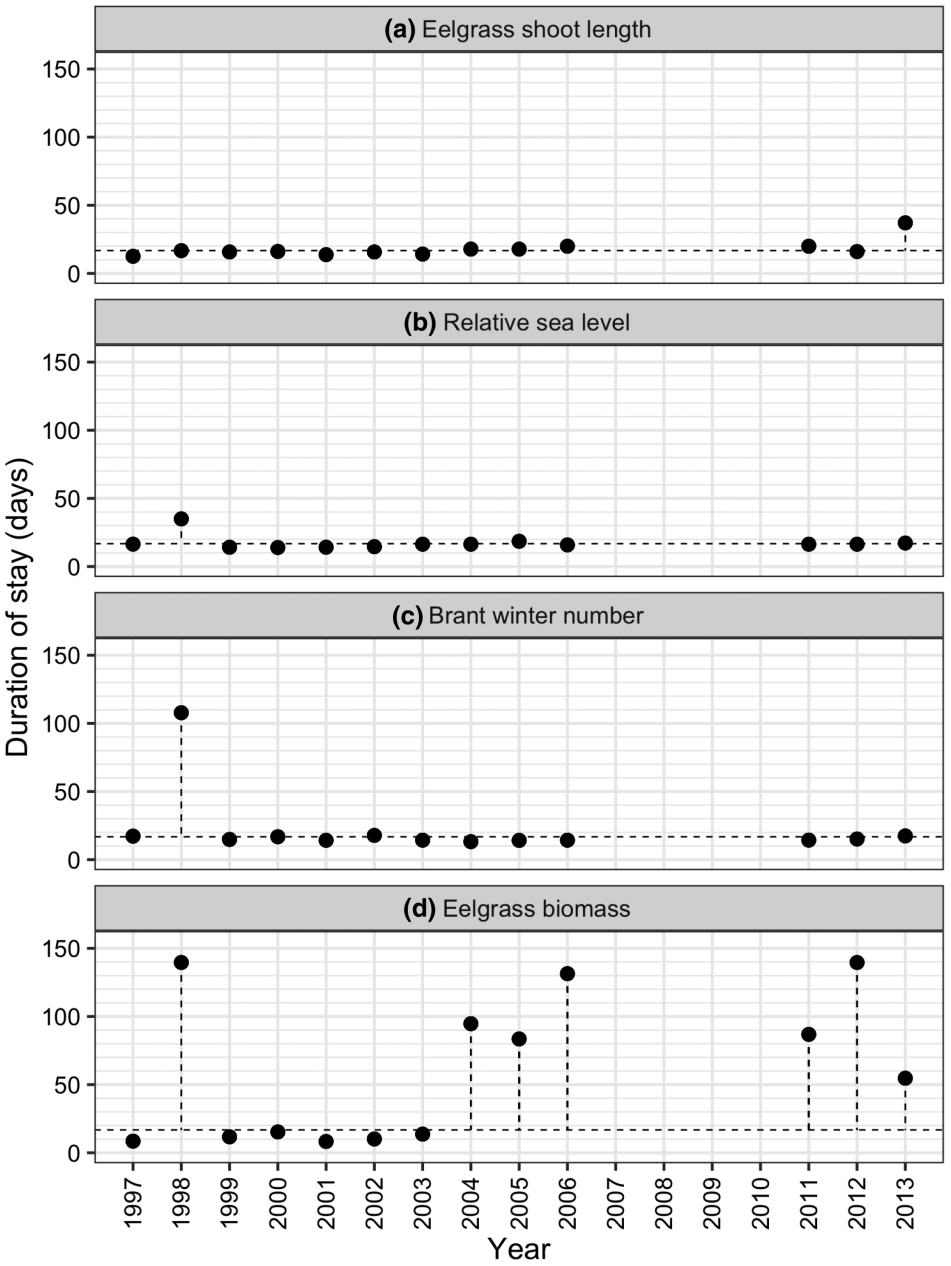
Predicted effect of between‐year variation in single variable simulations of (a) January eelgrass shoot length, (b) relative sea level, (c) brant winter population size, and (d) January eelgrass biomass on predicted duration of stay of spring migrants. Each figure shows year‐specific variability in predictions (solid symbols) of one parameter with values of the remaining three parameters held at their mean value across the years. The broken horizontal line shows the predicted duration of stay of spring migrants with all four parameters held at their mean value. The extent to which symbols deviate from the horizontal line indicates the amount to which the predicted duration of stay was dependent on the year‐specific value of a parameter.

There were small differences in the single variable simulations of eelgrass shoot length (Figure 7a) and sea level (Figure 7b) effects on predicted duration of stay of spring migrants, indicating that year‐to‐year variation in the predicted duration of stay was not strongly related to year‐to‐year variation in these parameters. For brant numbers, except for 1998, the difference was also relatively small and had little influence on predicted duration of stay (Figure 7c). Brant numbers were exceptionally high during the extreme ENSO winter of 1998 and only influenced predicted duration of stay in that 1 year. Eelgrass biomass, however, had a strong effect (high values) on predicted duration of stay in years when eelgrass biomass was low (Figure 7d).

### 3.3 Observed relationships between eelgrass biomass and brant body mass and survival

**FIGURE 8:**
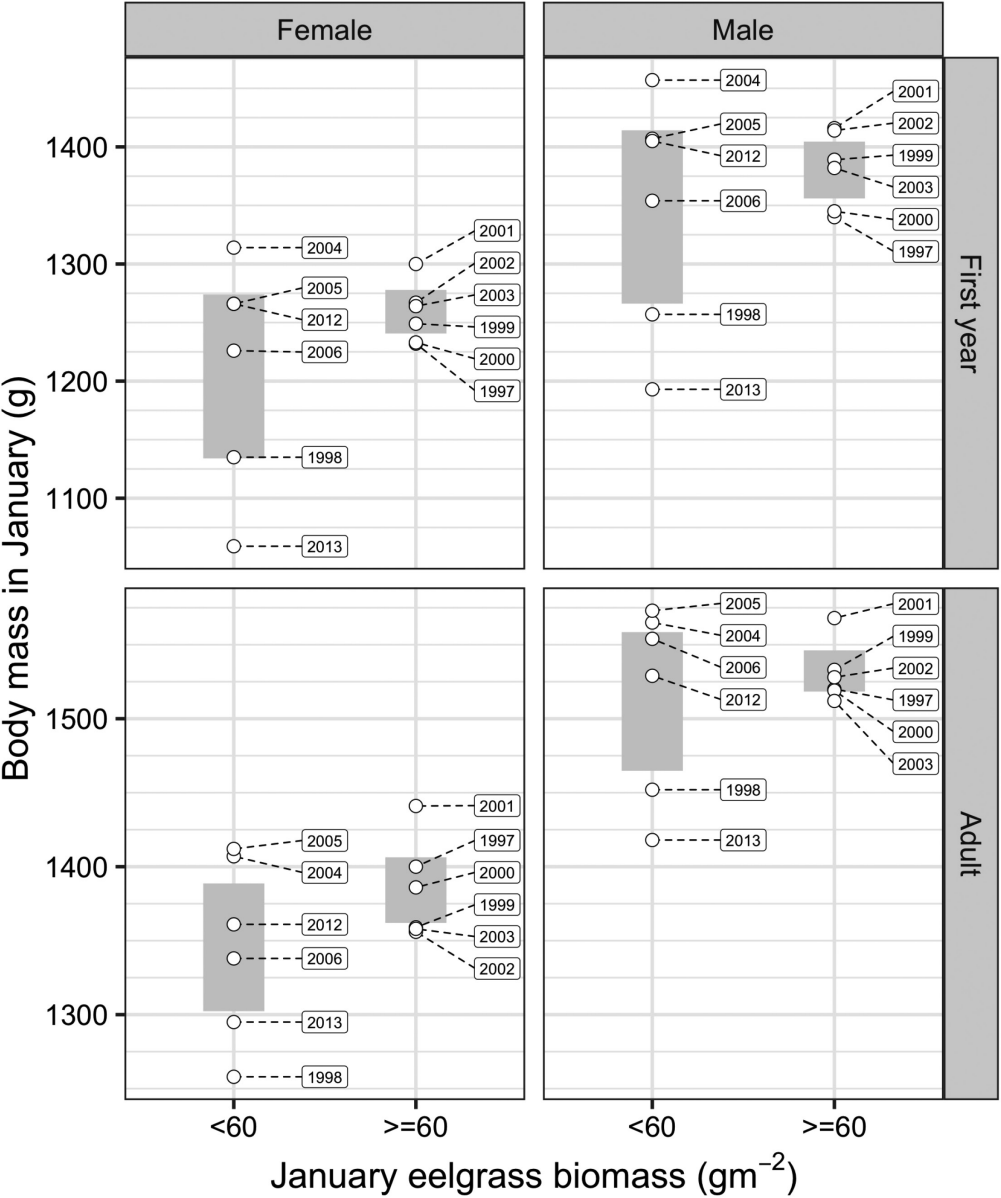
Observed body mass of brant age and sex combinations in years of low (<60 g m^−2^) and high (>60 g m^−2^) eelgrass biomass in Bahía San Quintín during January (except 2 of 8 sites sampled in 2012 during December). The gray bars show the 95% confidence intervals of mean body mass.

**FIGURE 9:**
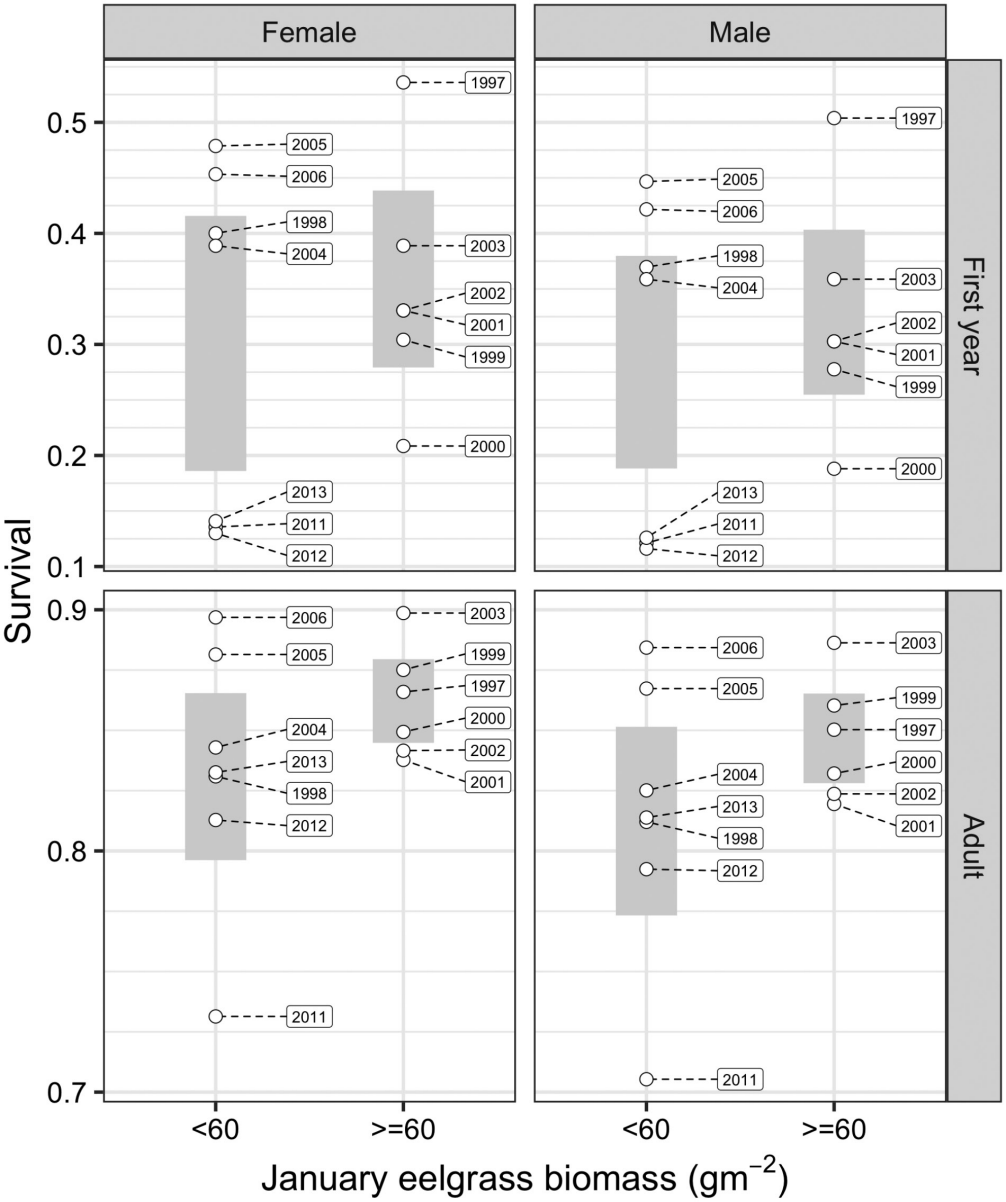
Observed annual survival brant age and sex combinations in years of low (<60 g m^−2^) and high (>60 g m^−2^) eelgrass biomass during January (except 2 of 8 sites sampled in 2012 during December). The gray bars show the 95% confidence intervals of mean annual survival.

The single variable simulations showed that annual variation in the predicted duration of stay of spring migrants was most strongly related to eelgrass biomass. The predicted duration of stay was greatest in 7 of 13 years, years in which January eelgrass biomass was less than 60 g m^−2^ (Figure 4c), as birds had more difficulty gaining body mass during these years. In all other years, January eelgrass biomass was greater than 60 g m^−2^. Therefore, we used a January eelgrass biomass of less than 60 g m^−2^ to indicate years in which birds would be predicted to have more difficulty maintaining their body mass and surviving (Figures 8 and 9). The lowest observed values for both body mass and survival were in years in which eelgrass biomass at Bahía San Quintín was lower than 60 g m^−2^. Mean body mass did not differ significantly between lower (<60 g m^−2^) and higher (≥60 g m^−2^) biomass years for any age/sex combination (Welch T‐test *p*‐values: first‐year female = 0.296; first‐year male = 0.444; adult female = 0.218; adult male = 0.611). Similarly, mean survival did not differ significantly between lower and higher biomass years for any age/sex combination (Welch T‐test *p*‐values: first‐year female = 0.555; first‐year male = 0.567; adult female = 0.234; adult male = 0.235). Between year variation in body mass was greater for first‐year birds, and adult males, but not adult females in lower biomass compared to higher biomass years (Bartlett Test for homogeneity of variances *p*‐values: first‐year female = 0.012; first‐year male = 0.028; adult female = 0.211; adult male = 0.023). In contrast, between year variation in survival did not differ significantly between lower and higher biomass years for any age/sex combination (Bartlett Test for homogeneity of variances *p*‐values: first‐year female = 0.394; first‐year male = 0.421; adult female = 0.082; adult male = 0.087).

## 4 DISCUSSION

This paper shows the importance of Bahía San Quintín as a wintering site for brant in Mexico, during a period of decline in the overall size of the Mexican wintering population, and annual fluctuations in environment factors (sea level rise, January eelgrass shoot length and biomass decline) and brant demographic factors (population size variation) that could adversely affect the birds. Our model predicted that the annual variation in eelgrass biomass was the major factor determining the ability of the birds to gain enough energy to migrate in spring. In contrast, sea level height and eelgrass shoot length had relatively minimal impact on the propensity to migrate. The local population size of brant in Bahía San Quintín had a negative impact only in the 1 year (1998) in which bird numbers were exceptionally high, during which depletion of the eelgrass food supply by the birds themselves was sufficiently large to reduce emigration rate. Comparison of model predictions to observations indicated that the lowest brant body mass and survival occurred primarily in years with low eelgrass biomass (<60 g m^−2^ in January) which occurred in 54% of the study years.

The different environmental factors included within the model affected the rate at which energy was assimilated by brant. Higher sea level and reduced eelgrass shoot length tended to reduce energy assimilation rate as eelgrass fell below the water's surface and was more likely to be out of reach of the birds. Increased brant population size tended to reduce energy assimilation rate due to increased depletion of eelgrass biomass from consumption by the birds, which then reduced abundance and availability of foods for the birds (i.e., exploitative competition). Even so, eelgrass biomass was predicted to have the greatest impact on the ability of the birds to migrate from Bahía San Quintín as it varied considerably more than other factors between years and had a direct effect on the rate at which energy was assimilated. The Humboldt Bay and Izembek Lagoon models (Stillman et al., 2015, 2021), on which the Bahía San Quintín model was based, similarly predicted that the overall biomass of food available is a key factor related to the survival and emigration ability of the birds.

As with any model, the model used in this study was a simplification of the real system. In particular, the model assumed that the only food resource available to the birds was eelgrass. Although eelgrass is the primary food for brant at Bahía San Quintín, and throughout the migratory route of this species (Ward et al., 2005), brant are known to consume other intertidal foods, such as the seaweed *Ulva* and the seagrass *Ruppia*, at times when the biomass of eelgrass is low or unavailable (Ward et al., 2003, 2005). Biomass of these alternative foods can vary widely across years depending on changes in water temperature (*Ruppia*; Johnson et al., 2003) or nutrients (*Ulva*; Zertuche‐González et al., 2009). Availability of *Ulva* and *Ruppia* was not included as environmental factors in the Bahía San Quintín model because we lacked sufficient data to determine their biomass, distribution, and consumption rates in the years simulated. The predictions of the model should therefore be interpreted as the impact of eelgrass biomass on the ability of the birds to survive and migrate from the site, regardless of any possible impacts of alternative food supplies. In years in which the model predicted that the eelgrass biomass was insufficient to support the birds, in the real system, the abundance of alternative food resources may have allowed a higher proportion of birds to survive and migrate successfully than was indicated by the model.

Although eelgrass biomass was predicted to be the major factor influencing the birds, for one year, 1998, the model predicted that local population size of brant influenced the birds' ability to migrate. This occurred because of the very high numbers of brant during this year, which increased exploitative competition on food in the model due to consumption of eelgrass by the birds. This, in turn, reduced the rate at which brant assimilated energy, and negatively impacted their ability to gain enough energy to migrate successfully. The increase of brant in Bahía San Quintín was an extreme natural example of the general northward shift in brant winter distribution during low food abundance (Ward et al., 2005). The 1998 ENSO was one of the most powerful ENSO events ever recorded (Paek et al., 2017) that was associated with a region‐wide increase in sea temperatures and sea level and declines in eelgrass abundance (Cabello‐Pasini et al., 2002; Echavarria‐Heras et al., 2006; Johnson et al., 2003). In Bahía San Quintín, eelgrass biomass decreased by 75% and sea level rose 12 cm from previous year levels (Figure 4a,c). Conditions were likely even more severe for the 60% of Mexican brant population wintering at bays farther south in Baja California, where compared to Bahía San Quintín, eelgrass is already less available (grows lower intertidally) and 3‐4x less abundant (Cabello‐Pasini et al., 2003; Ward, 2022b). Brant that wintered in these southerly areas moved back north (Lindberg et al., 2007) consistent with eelgrass availability as a key driver of brant use of nonbreeding areas (Moore & Black, 2006; Wilson & Atkinson, 1995).

The long‐term decline in eelgrass biomass in Bahía San Quintín likely continues to perpetuate the northward shift of brant from Mexico (Palacios & Heredia, 2021). Reduction of eelgrass biomass was severe in Bahía San Quintín (75% decline, 2001–2013; Figure 4c) and in other brant wintering areas in Baja California (Ward, 2022b) and southern California (Walter et al., 2020). As such, low eelgrass abundance in winter is also likely contributing to long‐term reductions in both the brant nesting population on YKD (Sedinger et al., 2020; Wilson, 2019) and productivity of the overall population (Ward et al., 2018). Losses of eelgrass in the southern portion of its range appear linked to increasing sea surface temperatures and storm/flood events (Walter et al., 2020; Ward et al., 2003). This is concerning given that ENSO events are projected to intensify and occur more frequently in the northeast Pacific under climate warming (Anderson, 1992; Bromirski et al., 2013; Cai et al., 2021), increasing future likelihoods of greater eelgrass losses in Mexico and increased northward shift in winter distribution of brant and decreased population size of brant on the YKD.

The model predicted a threshold January eelgrass biomass of 60 g m^−2^ below which birds were unable to successfully migrate from Bahía San Quintín by consuming eelgrass alone. Body mass and survival tended to be relatively low when January eelgrass biomass was below 60 g m^−2^, supporting the model prediction that birds would struggle maintaining or increasing energy reserves. Inconsistencies, however, did exist between the annual comparisons, such as in 2004 and 2005 when body mass and survival were high in years of low eelgrass biomass across age and sex classes (Figures 8 and 9). These outcomes could be explained by increased availability of *Ruppia* and *Ulva* in those years. We unexpectedly did not detect a difference in body mass of birds between low and high biomass years. We are uncertain for the primary reason of this difference, but the lack of significant difference could be explained, in part, by hunters unintentionally harvesting brant in greater body condition during years of low eelgrass biomass. Sport hunting of brant occurs primarily at shoreline grit site locations in Bahía San Quintín where families with juveniles concentrate (Ward, 2024; Ward et al., 1997) and members of this family status are socially dominant and have greater body mass and condition than other brant (Poisbleau et al., 2006).

Brant were predicted to struggle in 7 of 13 years spread over the 17‐year study with impacts on brant survival and body condition differing between age classes. Adult annual survival was relatively high (ca. 85%) and stable over similar years of this study (Leach et al., 2017; Sedinger et al., 2006), suggesting that adults were able to compensate in years of low eelgrass abundance to gain energy reserves for migration obtained from alternative foods. In contrast, first‐year brant, a group that already have a lower survival rate than adults (Leach et al., 2017), incurred even lower (from 46% to 26%) survival during years of low eelgrass abundance (Sedinger & Nicolai, 2011). This negative trend in first‐year survival was likely attributable to increased natural mortality from food limitations in winter and not to differential harvest from hunting because adult survival and band recovery rates were stable over the same period (Leach et al., 2018; Sedinger & Nicolai, 2011).

The model predicted that brant would have the most difficulty surviving and migrating from Bahía San Quintín when January eelgrass biomass was below 60 g m^−2^ especially if this coincided with a high local population size of brant. Due to lack of suitable data, the model did not include alternative food resources potentially exploited by brant when eelgrass biomass is low, and so further research to quantify the abundance and food value of such resources, especially in relation to variation in eelgrass biomass, would be an important step to increase the realism of a future model. The decline in eelgrass abundance appears to be driven by both climate warming (increasing sea temperature and precipitation) and other anthropogenic (sediment loading, mariculture expansion) causes (Ward et al., 2003). Management actions that can increase eelgrass abundance (e.g., seagrass restoration) in combination with reductions in sport harvest, which has increased since the years of this study (Leach et al., 2018), and human disturbance will be key to reversing, or at least stabilizing, the decline of the brant population in Mexico. We encourage the continuation of surveys to monitor population size of brant (Palacios & Heredia, 2021), sport harvest of brant, and the abundance (biomass, distribution) of eelgrass and alternative foods (Ward, 2022a, 2022b) in Mexico. Monitoring in Bahía San Quintín will be important because of its significance to brant in Mexico and where most of the harvest of these birds occurs in the flyway.

## AUTHOR CONTRIBUTIONS

**R. A. Stillman:** Conceptualization (equal); formal analysis (lead); methodology (equal); software (lead); writing – original draft (equal). **E. M. Rivers:** Conceptualization (supporting); data curation (supporting); formal analysis (supporting); methodology (supporting); writing – original draft (supporting). **W. Gilkerson:** Conceptualization (supporting); data curation (supporting); formal analysis (supporting); methodology (supporting); writing – original draft (supporting). **K. A. Wood:** Conceptualization (supporting); formal analysis (supporting); methodology (supporting); writing – original draft (supporting). **P. Clausen:** Conceptualization (supporting); data curation (supporting); methodology (supporting); writing – original draft (supporting). **C. Deane:** Data curation (supporting); writing – review and editing (supporting). **D. H. Ward:** Conceptualization (equal); data curation (lead); formal analysis (equal); funding acquisition (lead); methodology (equal); writing – original draft (equal).

## Supporting information


Appendix S1.

Appendix S2.

## Data Availability

The data that support the findings of this study are openly available in the following repositories: Ward (2024). Data from black brant (Branta bernicla nigricans) overwintering in three lagoons along the Baja California Peninsula, Mexico (ver 2.0, January 2024): U.S. Geological Survey data release, https://doi.org/10.5066/F7T43R88. Ward (2022a). Point sampling data for eelgrass (Zostera marina) and widgeongrass (Ruppia marina) abundance in embayments of the north Pacific coast of Baja California, Mexico, (ver 2.0, April 2023): U.S. Geological Survey data release, https://doi.org/10.5066/P9H4LBP3. Ward (2022b). Abundance of eelgrass (Zostera marina) at key Black Brant (Branta bernicla nigricans) wintering sites along the northern Pacific coast of Baja California, Mexico. U.S. Geological Survey Open‐File Report 2022–1078, 15 p., https://doi.org/10.3133/ofr20221078.
