# Supplementary material for: Predicting the response of a long‐distance migrant to changing environmental conditions in winter
Source: Ecol Evol. 2024 Jun 29;14(7):e11619. doi: 10.1002/ece3.11619 (PMC11214018; doi:10.1002/ece3.11619)
Supplement: Supplementary file 1 — Appendix S1. [file ECE3-14-e11619-s002.pdf]

APPENDIX S1

Predicting the response of a long-distance migrant to changing environmental conditions in winter

R.A. Stillman[1†], E.M. Rivers[1], W. Gilkerson[2], K.A. Wood[3], P. Clausen[4], C. Deane[5] & D.H. Ward[6]

[1] Department of Life and Environmental Sciences, Faculty of Science and Technology, Bournemouth University, Poole, Dorset, BH12 5BB, United Kingdom.

[2] Merkel & Associates, Inc., 5434 Ruffin Rd, San Diego, CA 92123, United States of America.

[3] Wildfowl & Wetlands Trust, Slimbridge, Gloucestershire, GL2 7BT. United Kingdom.

[4] Department of Ecoscience - Wildlife Ecology, Aarhus University, C.F. Møllers Allé 8, 8000 Aarhus C, Denmark.

[5] Department of Biology and Wildlife, University of Alaska Fairbanks, Fairbanks, Alaska 99775, United States of America.

[6] U.S. Geological Survey, Alaska Science Center, 4210 University Drive, Anchorage, AK 99508-4626, United States of America.

Appendix S1: Description of the Bahía San Quintín brant model

Modelling time, space, and environmental variables

The model simulated 288 days, from 1 August to 15 May, encompassing the period of brant usage of the site (Table 1a). The model defined 'fall' as 1 August to 15 December, 'winter' as 16 December to 15 February, and 'spring' as 16 February to 15 May (Table 1a), corresponding to the periods of brant fall-staging, overwintering, and spring-staging respectively. Time was divided into discrete 1-hour time steps, within which environmental conditions were assumed to remain constant. The model incorporated the diurnal cycle, with hours of daylight or night based on predicted times of civil twilight at sunrise and sunset derived from www.timeanddate.com for Ensenada, Mexico during 2016/17 (the closest location for which predictions were available, approximately 170 km north of the study site) (Table 1a). The model also incorporated the lunar cycle, with the moon illumination (proportion of full moon) during each night derived from www.timeanddate.com also for Ensenada during 2016/17 (Table 1a). Default tidal height (relative to mean lower low water; m mllw) (actual tidal height varied between simulation years – see main paper for details) during each time step was from San Diego (Broadway), USA during 2016/17. No continuous hourly tidal data were available for the study site but timing of tides and tidal heights from San Diego (Broadway) closely match those of the study site. Figure 1 shows a comparison of the tidal height at Bahía San Quintín and San Diego during a week period when tidal heights were measured at Bahía San Quintín. The mean difference in hourly tide range between the two sites was 3.7 cm (greater change in Bahía San Quintín). The timing of daily tide highs and lows from San Diego was within 30 minutes of the equivalent tide highs and lows measured at Bahía San Quintín.

The model included 3 subsites, termed Bahía Falsa, East Bay and Back Bay (see Table 2 for details). Across all subsites, the model represented space as a grid of 500x500m patches, each with an individual shore elevation (m mllw) (Table 1b; see Table 2 for details), and within each of which environmental conditions were assumed to be uniform. The water depth over each patch during each time step was calculated as the difference between tidal height during the time step, and the elevation of the patch (Table 1b). Patches were assumed to be exposed when the tidal height was lower than the elevation of the patch.

Modelling food resource biomass and shoot length

One potential food resource was included in the model, eelgrass, distributed across the 500x500m patches. Brant could potentially feed on eelgrass rooted within a patch (termed rooted eelgrass), or eelgrass that had become detached and was floating within a patch (termed floating eelgrass). Each patch had a fixed elevation (which determined its water depth through the tidal cycle), and potentially contained a specific aboveground biomass and shoot length of rooted eelgrass, which combined with elevation determined the availability of food for brant (see Table 2 for details).

The distribution of eelgrass in the model (i.e., the patches that were occupied) was derived from a combination of shore elevation, sediment type and the observed distribution of the species (Table 1b; see Table 2 for details). The biomass of eelgrass was set to zero in patches that were not occupied.

Initial rooted eelgrass aboveground biomass (gm^{-2}) and shoot length (m, meristem to tip of longest ungrazed leaf) were derived from bay-wide surveys using a systematic point-sampling design (Ward 2022a). Surveys were conducted during December between 1999 and 2012.

Quadratic regression was used to relate aboveground biomass to elevation (Table 1b; see Table 2 for details). Linear regression was used to relate default shoot length to elevation (Table 1b; see Table 2 for details). These relationships were then used to predict eelgrass December aboveground default biomass and shoot length relative to mean elevation (m mllw) of each patch (actual eelgrass biomass and shoot length varied between simulation years – see main paper for details). As eelgrass biomass and shoot length vary seasonally (see below and Table 1b for details), being greater before December, the initial values used in the model (for 1 August) were increased from the values recorded in December (Table 1b), such that the model values throughout the season matched those observed.

Changes in rooted eelgrass biomass from fall to spring were determined from monthly sampling of eelgrass (Figure 2a). This relationship was used to calculate rates of eelgrass change during two periods of the year, fall and winter biomass decline (1 November to 15 March), and spring and summer biomass increase (16 March to 1 June) (Table 1b). Eelgrass biomass was assumed to remain constant before 1 November and after 1 June. Changes in eelgrass shoot length through time were determined from seasonal changes between January 1999 and March 2000 (Cabello-Pasini et al. 2003) (Figure 2b). Relative eelgrass shoot length was calculated as a proportion of the annual maximum shoot length and a sine curve (to account for the seasonal cycling of shoot length) fitted to relative shoot length to determine the relationship between shoot length and day of the year (Table 1b).

Brant primarily feed on eelgrass rooted to the substrate when it is within reach at low tides but may also consume eelgrass that becomes displaced and floats to the water surface (e.g., Moore 2002, Elkinton et al. 2013). The biomass of floating eelgrass within each patch was assumed to be a fixed proportion of the biomass of rooted eelgrass (Table 1b). During each

time step, model birds could potentially feed on floating eelgrass, if this yielded a higher net energy intake rate (see below for details) than feeding on rooted eelgrass.

Modelling goose migration, energetics, and behaviour

The model considered three types of brant: fall migrants – birds that passed through the site during southward migration; spring migrants – birds that passed though the site during northward migration; and over-winterers – birds that spent the winter in the site. Although in the real system some fall and spring migrants may be the same geese, these birds were considered separately in the model as the model did not incorporate other staging and wintering sites to the south. Specific default numbers of fall migrants, over-winters and spring migrants were used in simulations (Table 1c) (actual number of over-winterers varied between simulation years – see main paper for details). Due to the large population size of brant in the site, rather than simulating each individual goose, model simulations used flocks comprised of 100 individuals. (i.e., 'super-individuals' *sensu* Scheffer et al., 1995). This assumption was realistic, as geese within sites tend to concentrate in large flocks rather than being spread individually throughout the sites (Ward 2024). Each model flock was randomly assigned a date when it arrived, drawn from a uniform distribution between the observed first and last arriving brant of each type (Table 1c). A uniform distribution was assumed, rather than an alternative type of distribution, for simplicity, and as limited data were available to determine the precise distribution of arrival dates.

The model tracked the amount of energy stored by each goose, calculated as body mass minus lean body mass, and multiplied by the energy content of fat (Table 1c). Geese of each type were also assigned a body mass at arrival in the system (Table 1c; Figure 3). Fall migrant geese were assumed to emigrate from the site a fixed number of days after their arrival,

irrespective of their body mass (Table 1c). Over-winterers and spring migrant geese were assumed to remain in the system until a specific departure day and departure energy store were reached (Table 1c; Figure 3).

Following Stillman et al. (2015), the energy expenditure of feeding and resting brant was calculated from multipliers of basal metabolic rate (Table 1c). Energetic costs due to thermoregulation were not included in the model, as temperature within the site is rarely below or above that at which the birds would need to thermoregulate (Mason et al. 2006). As feeding energy expenditure was greater than resting energy expenditure (Table 1c), the amount of energy expended by an individual within each one-hour time step depended on the proportion of the time step spent feeding and resting.

The model assumed that rooted eelgrass was available to the geese if within the maximum foraging depth of brant (Table 1c). For each time step, the water depth to eelgrass was calculated as the difference between water level and the elevation of the patch plus the eelgrass shoot length. Following Stillman et al. (2021), this assumed that the plant shoots were standing upright in the water column and did not bend due to water current. Rooted eelgrass was classed as either fully available to geese, or not available to geese, depending on whether it was within reach of the water surface or exposed by the tide (Table 1c). Floating eelgrass was assumed to be always available to the geese.

Following Stillman et al. (2021), the rate at which the model geese could consume eelgrass was calculated using a functional response, relating the biomass of food to the rate of consuming food, as derived from light-bellied brent geese (*Branta bernicla hrota*) feeding on *Z. marina* in Europe (Clausen 2013) (Table 1c). The model assumed that the rate of feeding (for a fixed biomass) was the same during daylight and moonlit nights and geese fed on either

rooted or floating eelgrass, depending on which resource type maximised their rate of energy assimilation.

The model incorporated competition due to resource depletion, with each model flock depleting 100 times the amount of eelgrass consumed by an individual goose. The biomass of food available to birds within a patch during a time step depended on the previous depletion through consumption by the birds and seasonal changes in biomass due to other factors (Table 1b). Resource competition occurred both within and between simulated flocks through the successive depletion of eelgrass between time steps. Other types of competition, such as aggression or competitor avoidance, were not modelled explicitly, as they were not considered important in this system, and would have been incorporated to some extent in the functional response as it was based on observations of real geese. No limit was set on the number of geese that could potentially occur in a single patch of eelgrass.

The rate at which model geese could assimilate energy from their food was calculated from the rate at which they could feed, the energy content of the resource and their digestive efficiency in assimilating the resource (Table 1c). A limit was placed on the maximum possible daily intake of energy, based on an allometric equation following Kirkwood (1983) (Table 1c).

Model geese had a season-dependent target size of energy (e.g., fat) store that they attempted to achieve (Table 1c; Figure 3). During fall, fall migrants and over-winterers attempted to achieve the fall target energy store size. During winter, over-winterers attempted to achieve the winter target energy store size. During spring, and from a fixed date, over-winterers and spring migrants attempted to achieve the departure energy store size (i.e., the energy store size required to migrate northward from the site). Before this date, over-winterers and spring migrants attempted to achieve the winter target energy store size.

On reaching their target energy store, model geese regulated the amount of food they consumed by reducing the proportion of time they spent feeding within each time step, and hence increasing resting time. However, if model geese were unable to meet their energy requirements, they drew energy from their store, thus decreasing overall size of the store. For simplicity, over-winterer and spring migrant geese that could not reach their departure energy store remained in the system. If a bird's energy store fell to zero, it died of starvation. Therefore, mortality due to starvation was incorporated as an all-or-nothing response to reduction in energy store size, in which birds survived if the size of their energy store was greater than zero but died as soon as the store size fell to zero. As the primary purpose of the model was to predict the effect of food abundance, availability, and competition on brant, direct sources of mortality through hunting or predation were not incorporated.

During each time step, model geese moved to the patch and consumed the resources (i.e., either rooted or floating eelgrass) which maximised their net rate of energy consumption (measured as energy assimilation divided by energy expenditure), taking account of energy assimilation, and energy costs of foraging and resting. When selecting a patch to occupy, the model birds did not consider the overall size of a continuous patch of eelgrass (i.e., the number of neighbouring patches containing eelgrass), instead, their decisions were based on the food supply available within each individual patch. Model birds did not necessarily stay within an individual patch for a number of times steps, instead, they would move between successive patches during successive time steps, if the patch which maximised the net rate of energy consumption changed. The distance and energetic cost of moving between patches was not incorporated into the model.

Year-specific parameter values used in simulations

Simulations were run to predict the effect on brant of annual variation in relative sea level, brant overwinter population size, eelgrass biomass and shoot length for 13 years, 1997–2006 and 2011 –2013. In these simulations, all other parameter values were assumed to remain unchanged, with values as in Table 1. Values of relative sea level, brant overwinter population size, eelgrass biomass and shoot length were adjusted from the default values in Table 1 to match the values observed each year. Table 3 lists the adjustments used for each year. Relative sea level each year was added to the default sea level for each model time step. This either increased or decreased water level, depending on whether the relative sea level for a year was positive or negative. For brant overwinter population size, the observed overwinter population size was used for each year, by multiplying the default population size by a year-specific value. Eelgrass biomass and shoot length varied between model patches depending on its shore elevation, season, and year. The model was initially run using the default parameter values of eelgrass biomass and shoot length listed in Table 1. The mean values of predicted eelgrass biomass and shoot length were then calculated during January within a shore elevation range of -1.0 to -0.3 m mllw (i.e., the month and shore elevations from which the observed data were collected). For eelgrass shoot length, the year-specific adjustment was the ratio of the observed January shoot length and the shoot length predicted by the default parameter values. The model was then re-run with these year-specific adjustments to confirm that the predicted January shoot length each year matched the observed. For eelgrass biomass, a similar process was used, but also needed to take account of depletion of eelgrass by the birds. The model was run both with the year-specific number of birds present and with no birds present to calculate the biomass of eelgrass consumed by the birds before

January. This made it possible to calculate the initial biomass of eelgrass required each year (and therefore the appropriate adjustment to default biomass), such that the combination of consumption by the birds and other sources of biomass decline, resulted in the year-specific January eelgrass biomass being equal to the observed.

Acknowledgements

Any use of trade, firm, or product names is for descriptive purposes only and does not imply endorsement by the U.S. Government.

References

Cabello-Pasini, A., R. Muñiz-Salazar and D. H. Ward 2003. Annual variations of biomass and photosynthesis in *Zostera marina* at its southern end of distribution in the North Pacific. Aquatic Botany 76: 31-47.

Clausen, K. K. 2013. Climate change impacts on coastal herbivorous waterbirds. PhD Dissertation. Aarhus University, Aarhus, Denmark.

Clausen, P. 1994. Waterfowl as primary consumers in shallow water fiord areas. PhD Dissertation, University of Aarhus/National Environmental Research Institute, Aarhus, Denmark.

Clausen, P. 2000. Modelling water level Influence on habitat choice and Food Availability for Zostera Feeding Brent Geese *Branta bernicla* in Non-Tidal Areas. Wildlife Biology 6: 75–87.

Clausen, K. K., P. Clausen, C. C. Fælled and K. N. Mouritsen. 2012. Energetic consequences of a major change in habitat use: endangered brent geese *Branta bernicla hrota* losing their main food resource. Ibis 154: 803-814.

Elkinton, E., L. Lo, and J. M. Black. 2013. Black brant Branta bernicla nigricans forage at both tides on Humboldt Bay, California, USA. Wildfowl Special Issue 3: 90–103.

Kersten, M., and T. Piersma. 1987. High levels of energy expenditure in shorebirds: metabolic adaptations to an energetically expensive way of life. Ardea 75: 175–187.

Kirkwood, J. K. 1983. A limit to metabolizable energy intake in mammals and birds. Comparative Biochemistry and Physiology Part A: Molecular & Integrative Physiology 75: 1–3.

Mason, D. D., Barboza, P. S., & Ward, D. H. (2006). Nutritional condition of Pacific Black Brant wintering at the extremes of their range. The Condor, 108: 678-690.

Mason, D. D., Barboza, P. S., & Ward, D. H. (2007). Mass dynamics of wintering Pacific Black Brant: Body, adipose tissue, organ, and muscle masses vary with location. Canadian Journal of Zoology, 85: 728–736.

Moore, J. E. 2002. Distribution of spring staging black brant Branta bernicla nigricans in relation to feeding opportunities on south Humboldt Bay, California. MSc Thesis. Humboldt State University, Arcata, California, USA.

Scheffer, M., Baveco, J. M., DeAngelis, D. L., Rose, K.A., & van Nes, E. (1995). Super-individuals a simple solution for modelling large populations on an individual basis. Ecological Modelling, 80: 161-170.

Spaans, B., K. van't Hoff, W. van der Veer, and B. S. Ebbinge. 2007. The significance of female body stores for egg laying and incubation in dark-bellied brent geese *Branta bernicla bernicla*. Ardea 95: 3–15.

Stillman, R.A., Wood, K. A., Gilkerson, W., Elkinton, E., Black, J. M., Ward, D. H. and Petrie, M. 2015. Predicting effects of environmental change on a migratory herbivore. Ecosphere 6(7): 114. http://dx.doi.org/10.1890/ES14-00455.1.

Stillman, R.A., Rivers, E. M., Gilkerson, W., Wood, K. A., Nolet, B. A., Clausen, P., Wilson, H. M. and Ward, D. H. 2021. Predicting impacts of food competition, climate, and disturbance on a long-distance migratory herbivore. Ecosphere 12(3): e03405. https://doi.org/10.1002/ecs2.3405.

Ward, D. H. (2022a). Abundance of Eelgrass (Zostera marina) at key Black Brant (Branta bernicla nigricans) wintering sites along the northern Pacific coast of Baja California, Mexico. U.S. Geological Survey Open-File Report 2022–1078, 15 p., https://doi.org/ 10.3133/ ofr20221078.

Ward, D. H. (2022b). Point sampling data for eelgrass (*Zostera marina*) and widgeongrass (*Ruppia maritima*) abundance in embayments of the north Pacific coast of Baja California, Mexico, 1997-2013: U.S. Geological Survey data release, https://doi.org/10.5066/P9H4LBP3.

Ward, D. H., (2024). Data from black brant (*Branta bernicla nigricans*) overwintering in three lagoons along the Baja California Peninsula, Mexico (ver 2.0, January 2024): U.S. Geological Survey data release, https://doi.org/10.5066/F7T43R88.

Table 1. Parameter values used in the model.

(a) Time and environmental parameters

Parameter	Value(s)	Derivation
Time step length (hours)	1	Time during which environmental conditions are assumed to remain constant.
Model duration (days)	288	1 Aug to 15 May. Encompassing period of usage of study site by brant.
Seasons	Fall = 1 Aug – 15 Dec<br>Winter = 16 Dec – 15 Feb<br>Spring = 16 Feb – 15 May	Based on duration of stay of migrant brant in fall, overwintering brant in winter, and migrant brant in spring.
Duration of daylight (hours)	11.0 – 14-7	Range of daylight hours (twilight to twilight) from 1 Aug 2016 to 15 May 2017. Derived for Ensenada, Mexico (closest location for which predictions were available) from www.timeanddate.com.
Moon illumination during night (proportion)	0 – 1	Proportion of full moon each night from 1 Aug 2016 to 15 May 2017. Derived for Ensenada, Mexico from www.timeanddate.com.
Water level (m mllw)	-0.52 – 2.29	Hourly tidal water level data for 1 Aug 2016 to 15 May 2017 from San Diego (Broadway), USA (https://www.tidesandcurrents.noaa.gov/). No hourly tidal data were available for the study site but tidal heights from San Diego (Broadway) closely match these (Figure 1).

(b) Patch and resource parameters

Parameter	Value(s)	Derivation
Patch size (m^2)	500x500 = 250000	Maximum area exploited by an individual in a single hour time step.
Number of patches	98	Total number of discrete 500x500 m patches occupied by eelgrass across all sites. See Table 2 for details.
Number of subsites	3 = Bahía Falsa, East Bay, Back Bay	Number of individual subsites. See Table 2 for details.
Patch elevation (m mllw)	-2.82 – 0.45	Derived from digital terrain model. Patch elevation calculated as the mean elevation within patch. See Table 2 for details.
Initial eelgrass biomass (B) (g DM m^{-2})	B = 0 if absent from patch<br>$B = (137 / 85) \times (17.90 - 26.89H - 9.1056H^2)$<br>where H = patch elevation (m mllw).	Relationship with elevation ($17.90 - 26.89H - 9.1056H^2$) derived from December surveys of eelgrass biomass during 1999 and 2012 (Ward 2022b). (137/85) converts December biomass to initial biomass. See Table 2 for details.

Parameter	Value(s)	Derivation
Seasonal change in eelgrass biomass	Start of biomass decline = 1 Nov (93 days from 1 Aug)<br>Proportional change per time step = 0.9997867533<br>End of biomass decline = 15 Mar (227 days from 1 Aug)<br>Start of biomass increase = 16 Mar (228 days from 1 Aug)<br>Proportional change per time step = 1.0003664530<br>End of biomass increase = 1 Jun (305 days from 1 Aug)	Changes in rooted eelgrass biomass from fall to spring determined from monthly sampling of eelgrass meadows (Cabello-Pasini et al. 2003) (Figure 2).
Initial eelgrass shoot length (L) (m)	L = (56 / 39) x (0.2477 – 0.1285H)<br>where H = patch elevation (m mllw).	Relationship with elevation derived from December surveys of eelgrass shoot length during 2005 to 2006 (Ward 2022b). See Table 2 for patch-specific values.
Seasonal changes in eelgrass shoot length (L_{rel})	L_{rel} = (23.10 + (36.52 x 0.5 x (1 + sin(2 x pi x ((52.03 + d) / 365))))) / (23.10 + 36.52)<br>where d = days from 1 Aug	Changes through time determined between January 1999 and March 2000 by Cabello-Pasini et al. (2003) (Figure 2). Relative shoot length calculated as proportion of annual maximum. Sine curve fitted to relative shoot length to determine relationship throughout the year.
Eelgrass metabolizability (%)	51	Proportion of energy within eelgrass that is assimilated by the geese. Derived for *Zostera marina* leaves. (Mason et al. 2006).
Eelgrass energy content (KJ g-1 DM)	14.15	Amount of energy per g of eelgrass. Derived for *Zostera marina* leaves at Bahía San Quintín; (Mason et al. 2006).
Eelgrass floating biomass (% of rooted biomass in patch)	5%	Floating eelgrass available throughout each season. Estimated to be the same as in Izembek Lagoon (Stillman et al. 2015) and Humboldt Bay (Stillman et al. 2021).

(c) Brant parameters

Parameter	Value(s)	Derivation
Population size	In fall = 31,461 (of which 9,866 during fall only and 21,595 during fall and winter)<br>In winter = 21,595<br>In spring = 13,977	Based on ground population surveys and length of stay estimates of migrating and wintering radio-marked brant (Ward 2024).
Size of flocks	100	Realistic as geese in site form large flocks (Ward 2024).
First and last arrival dates	Fall migrant = 15 Oct to 10 Dec<br>Over-winterer = 15 Oct to 15 Jan<br>Spring migrant = 11 Feb to 15 Apr	Based on ground population surveys and length of stay estimates of migrating and wintering radio-marked brant (Ward 2024).

Parameter	Value(s)	Derivation
Departure date	Fall migrant = 11 days after arrival irrespective of body mass<br>Over-winterer / Spring migrant = from 10 Feb if departure body mass reached	Based on radio telemetry tracking of radio-marked brant at Bahia San Quintin (Ward 2024).
Energy density of fat (KJ g-1)	34.3	Energy content of avian tissue (Kersten and Piersma 1987)
Starvation mass (g)	964	Spaans et al. (2007) estimated the lean mass (i.e., with no energy stores) of dark-bellied brent geese *Branta bernicla bernicla* to be 73% of mass on arrival at breeding area. Thus, for brant, 1320g * 0.73 = 964 g
Body mass on arrival (g)	Fall migrant = 1376<br>Over-winterer = 1376<br>Spring migrant = 1491	Derived from body masses of collected (Mason et al. 2007) and hunted (Ward 2024) birds (Figure 3). Fall migrant and over-winterer = body mass at start of fall. Spring migrant = mean of body mass during winter so that spring migrants initially have same mass as winter residents.
Energy store size on arrival (g)	Fall migrant = 14131.6<br>Over-winterer = 14131.6<br>Spring migrant = 18076.1	Calculated from:<br>Energy density of fat x (Body mass on arrival – Starvation mass)
Target body mass during staging (g)	In fall = 1430<br>In winter = 1491<br>In spring = 1586	Derived from body masses of collected (Mason et al. 2007) and hunted (Ward 2024) birds (Figure 3). In fall = body mass at end of fall. In winter = mean body mass during winter. In spring = body mass at end of spring.
Departure body mass (g)	Fall migrant = n/a<br>Over-winterer = 1586<br>Spring migrant = 1586	Derived from body masses of collected (Mason et al. 2007) and hunted (Ward 2024) birds (Figure 3). Fall migrant = no departure mass as depart after 11 days. Over-winterer / spring migrant = body mass at end of spring.
Target energy size (KJ)	In fall = 15983.8<br>In winter = 18076.1<br>In spring (to 14 Feb) = 18076.1<br>In spring (from 15 Feb) = 21334.6	During staging (i.e., until 14 Feb) calculated from:<br>Energy density of fat x (Target body mass during staging – Starvation mass)<br>During spring migration (i.e., from 15 Feb) calculated from:<br>Energy density of fat x (Departure body mass – Starvation mass)
Maximum foraging depth (m)	0.40	Taken from Clausen (1994, 2000).
Rate of consuming eelgrass biomass (g DM hr^{-1}) (C)	$C = 60\frac{0.419B}{20.2+B}$<br>where B = eelgrass biomass (g DM m^{-2}).	Calculated from consumption rates of East Atlantic light-bellied Brent goose (*Branta bernicla hrota*), feeding on eelgrass (*Zostera marina*; Clausen 2013).
Maximum energy assimilation (KJ day^{-1}) (E_{max})	$E_{max} = 1713(M/1000)^{0.72}$<br>$E_{max} = 2271$<br>where M = body mass (g) = 1479	Calculated from body mass using equation derived by Kirkwood (1983). Body mass is across-season mean derived from hunted birds (Ward 2024; Figure 3).

Parameter	Value(s)	Derivation
Basal Metabolic Rate (*BMR*) (J s^{-1})	In fall = 5.78<br>In winter = 6.05<br>In spring = 6.20	Calculated from mean mass (g) in fall (1397), winter (1491) and spring (1546) using allometric equation ($BMR = 4.59\ (\text{Mass} / 1000)^{0.69}$) (Bruinzeel et al. 1997). Body masses derived from collected (Mason et al. 2007) and hunted (Ward 2024) birds (Figure 3).
Energy expenditure while foraging (J s^{-1})	In fall = 1.9 x 5.78<br>In winter = 1.9 x 6.05<br>In spring = 1.9 x 6.20	Calculated as a multiplier (1.9) of *BMR* (Clausen et al. 2012).
Energy expenditure while resting (J s^{-1})	In fall = 1.6 x 5.78<br>In winter = 1.6 x 6.05<br>In spring = 1.6 x 6.20	Calculated as a multiplier (1.6) of *BMR* (Clausen et al. 2012).

Table 2. Patch-specific parameter values used in the model: Subsite number = Number of subsite in which patch is located (1 = Bahía Falsa; 2 = East Bay; 3 = Back Bay); Patch X coordinate (m) = X coordinate of center of patch (projected in NAD 83 UTM Zone 11 North); Patch Y coordinate (m) = Y coordinate of center of patch (projected in NAD 83 UTM Zone 11 North); Patch elevation (m) = Patch shore elevation (measured relative to Mean Lower Low Water (MLLW)); Eelgrass shoot length (m) = Initial eelgrass shoot length in patch, calculated from patch elevation (see Table 1b for formula; data from Ward (2022b)); Eelgrass biomass (g dry mass $^{-2}$) = Initial eelgrass biomass in patch, calculated from patch elevation (see Table S1b for formula; data from Ward (2022b)).

Patch number	Subsite number	Patch X coordinate (m)	Patch Y coordinate (m)	Patch elevation (m MLLW)	Eelgrass shoot length (m)	Eelgrass biomass (g dry mass $^{-2}$)
1	3	596938	3374038	0.38	0.286	10.53
2	3	597438	3373538	0.07	0.343	25.76
3	3	597438	3373038	0.09	0.340	24.99
4	3	597938	3373038	0.41	0.281	8.89
5	3	597938	3372538	-0.05	0.365	30.90
6	2	598938	3372538	-0.76	0.497	53.41
7	2	599438	3372538	0.32	0.297	13.50
8	2	598438	3372038	0.39	0.284	9.69
9	2	598938	3372038	-1.44	0.621	60.82
10	2	599438	3372038	-1.03	0.545	57.90
11	2	598438	3371538	0.40	0.282	9.27
12	2	598938	3371538	-1.06	0.551	58.27
13	2	599438	3371538	-2.70	0.855	38.72
14	2	599938	3371538	-0.34	0.418	41.86
15	2	599438	3371038	-1.46	0.624	60.84
16	2	600438	3371038	0.07	0.343	25.84
17	2	599938	3370538	-0.71	0.486	52.12
18	2	600438	3370538	0.26	0.307	16.39
19	2	599938	3370038	-2.82	0.876	34.31
20	2	600438	3370038	-0.47	0.443	46.07
21	2	600438	3369538	-0.96	0.533	56.93
22	2	600938	3369538	0.18	0.323	20.70
23	1	593938	3369038	-0.06	0.367	31.50
24	1	594438	3369038	-0.38	0.425	43.11
25	1	594938	3369038	-0.72	0.489	52.53
26	1	595438	3369038	-0.75	0.494	53.13
27	1	595938	3369038	-0.50	0.448	46.91
28	1	596438	3369038	-0.54	0.455	48.00
29	2	600938	3369038	0.27	0.306	16.25
30	1	594438	3368538	-0.34	0.419	42.00
31	1	594938	3368538	-0.77	0.497	53.45

Patch number	Subsite number	Patch X coordinate (m)	Patch Y coordinate (m)	Patch elevation (m MLLW)	Eelgrass shoot length (m)	Eelgrass biomass (g dry mass $^{-2}$)
32	1	595438	3368538	-0.79	0.502	53.97
33	1	595938	3368538	-0.53	0.454	47.83
34	1	596438	3368538	-0.51	0.450	47.14
35	2	600438	3368538	-0.81	0.505	54.35
36	2	600938	3368538	0.11	0.336	24.10
37	1	594938	3368038	-0.26	0.404	39.25
38	1	595438	3368038	-0.69	0.483	51.82
39	1	595938	3368038	-0.68	0.481	51.48
40	2	599938	3368038	-0.93	0.527	56.46
41	2	600438	3368038	-1.40	0.614	60.76
42	1	594938	3367538	0.01	0.353	28.28
43	1	595438	3367538	-0.89	0.520	55.82
44	1	595938	3367538	-0.99	0.538	57.36
45	1	596438	3367538	-1.67	0.663	60.31
46	1	596938	3367538	-0.84	0.510	54.81
47	2	599438	3367538	-0.41	0.431	44.13
48	2	599938	3367538	-1.65	0.659	60.43
49	2	600938	3367538	0.18	0.322	20.50
50	1	594938	3367038	0.34	0.292	12.22
51	1	595438	3367038	-0.55	0.456	48.12
52	1	595938	3367038	-0.71	0.487	52.27
53	1	596438	3367038	-0.75	0.495	53.15
54	1	596938	3367038	-1.99	0.724	56.92
55	2	599438	3367038	-0.32	0.415	41.24
56	2	600438	3367038	-0.49	0.446	46.61
57	2	600938	3367038	0.45	0.272	6.10
58	1	595438	3366538	-0.19	0.391	36.51
59	1	595938	3366538	-0.26	0.403	39.00
60	1	596438	3366538	-0.51	0.449	47.03
61	1	596938	3366538	-1.19	0.576	59.66
62	1	597438	3366538	-1.70	0.670	60.09
63	1	597938	3366538	-1.35	0.604	60.61
64	1	598438	3366538	-1.06	0.552	58.33
65	2	598938	3366538	-0.83	0.509	54.74
66	2	599438	3366538	-0.96	0.533	56.94
67	2	600438	3366538	-0.34	0.419	42.02
68	2	600938	3366538	0.28	0.303	15.39
69	1	595938	3366038	-0.05	0.364	30.87
70	1	596438	3366038	-0.39	0.428	43.56
71	1	596938	3366038	-0.06	0.367	31.44

Patch number	Subsite number	Patch X coordinate (m)	Patch Y coordinate (m)	Patch elevation (m MLLW)	Eelgrass shoot length (m)	Eelgrass biomass (g dry mass $^{-2}$)
72	1	597438	3366038	0.24	0.311	17.50
73	1	597938	3366038	-0.01	0.358	29.29
74	1	598438	3366038	-0.68	0.480	51.43
75	2	599938	3366038	-0.38	0.426	43.32
76	2	600438	3366038	-0.13	0.379	34.09
77	2	600938	3366038	0.24	0.312	17.64
78	1	595938	3365538	-2.46	0.809	46.76
79	1	596438	3365538	-0.24	0.399	38.25
80	1	596938	3365538	0.36	0.290	11.49
81	1	597938	3365538	0.37	0.287	10.78
82	1	598438	3365538	-0.75	0.494	53.10
83	2	599438	3365538	-0.53	0.453	47.70
84	2	599938	3365538	-0.08	0.370	32.18
85	2	600438	3365538	-0.06	0.367	31.37
86	2	600938	3365538	0.25	0.309	16.98
87	1	596938	3365038	-1.55	0.641	60.78
88	2	598938	3365038	-0.76	0.496	53.34
89	2	599438	3365038	0.29	0.302	15.10
90	2	599938	3365038	0.30	0.300	14.31
91	2	600438	3365038	0.32	0.297	13.62

Table 3. Year-specific parameter adjustments to simulate annual variation in relative sea level, brant overwinter population size, eelgrass biomass and shoot length. The value for relative sea level was added to sea level during each model time step to obtain year-specific sea levels (values below zero decreasing and values above zero increasing sea level). The values for brant overwinter population size, eelgrass biomass and shoot length were multiplied with default values to obtained year-specific values of these parameters (values below 1 decreasing and values above 1 increasing these parameter values). The bottom row is the mean adjustment calculated across all years.

Year	Relative sea level (m)	Brant overwinter population	Eelgrass biomass	Eelgrass shoot length
1997	-0.02	1.34	5.36	2.07
1998	0.10	3.30	1.91	1.11
1999	-0.06	1.16	3.75	1.44
2000	-0.07	1.33	3.00	1.15
2001	-0.06	0.94	5.25	1.64
2002	-0.05	1.31	4.48	1.44
2003	0.01	0.93	3.21	1.40
2004	0.01	0.92	2.30	0.91
2005	0.04	0.77	2.38	0.91
2006	-0.03	0.93	1.83	0.77
2011	0.01	0.86	1.46	0.77
2012	0.01	1.14	1.91	1.06
2013	0.02	1.35	1.14	0.48
**Mean**	**-0.01**	**1.25**	**2.92**	**1.17**

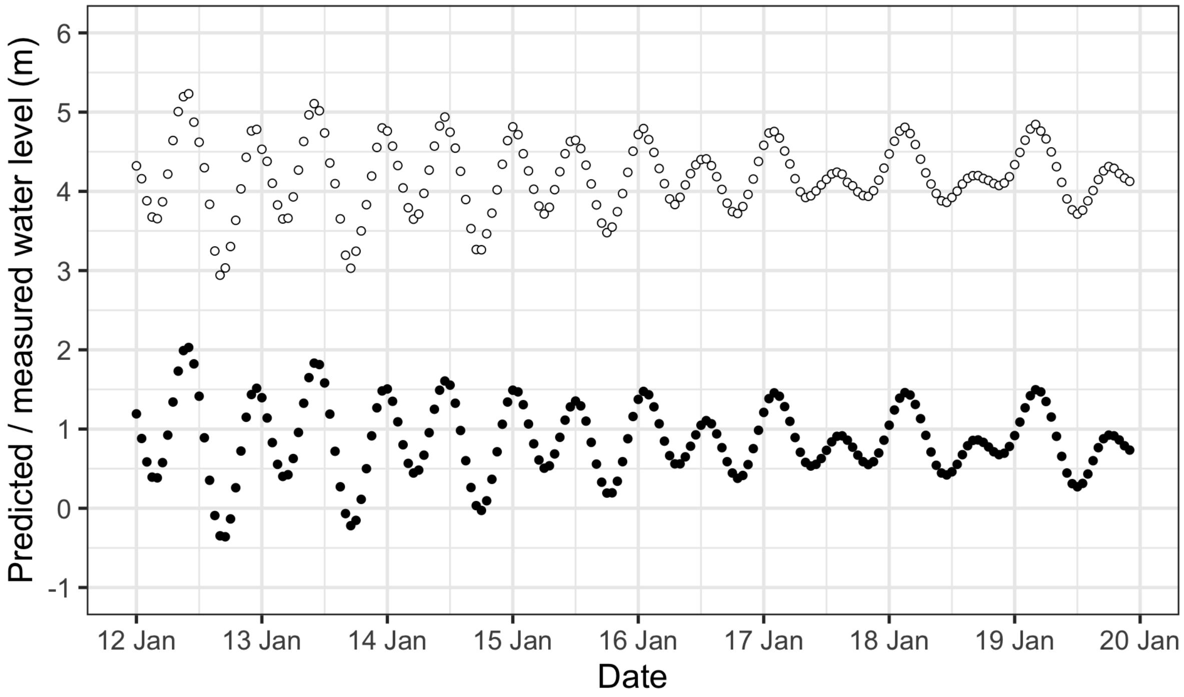

Figure 1. Comparison of hourly changes in predicted tide height at San Diego (solid symbols) against measured water level from a depth recorder in Bahía San Quintín (open symbols) over a week period from 12 January 2013 to 19 January 2013.

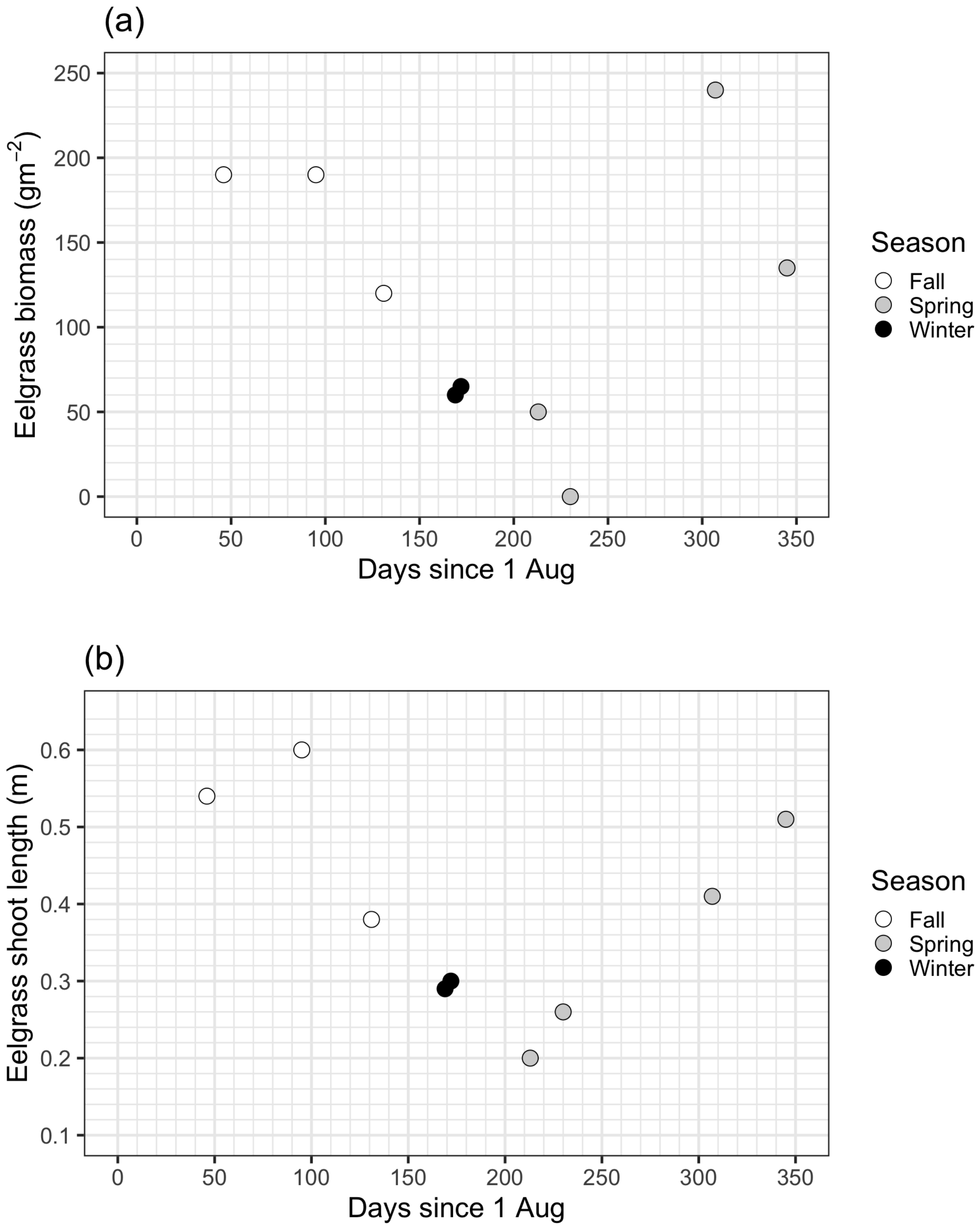

Figure 2. Seasonal changes in (a) eelgrass biomass and (b) eelgrass shoot length in Bahía San Quintín. Data from Figure 5 of Cabello-Pasini et al. (2003). The symbol shading indicates the dates of the seasons used in the model.

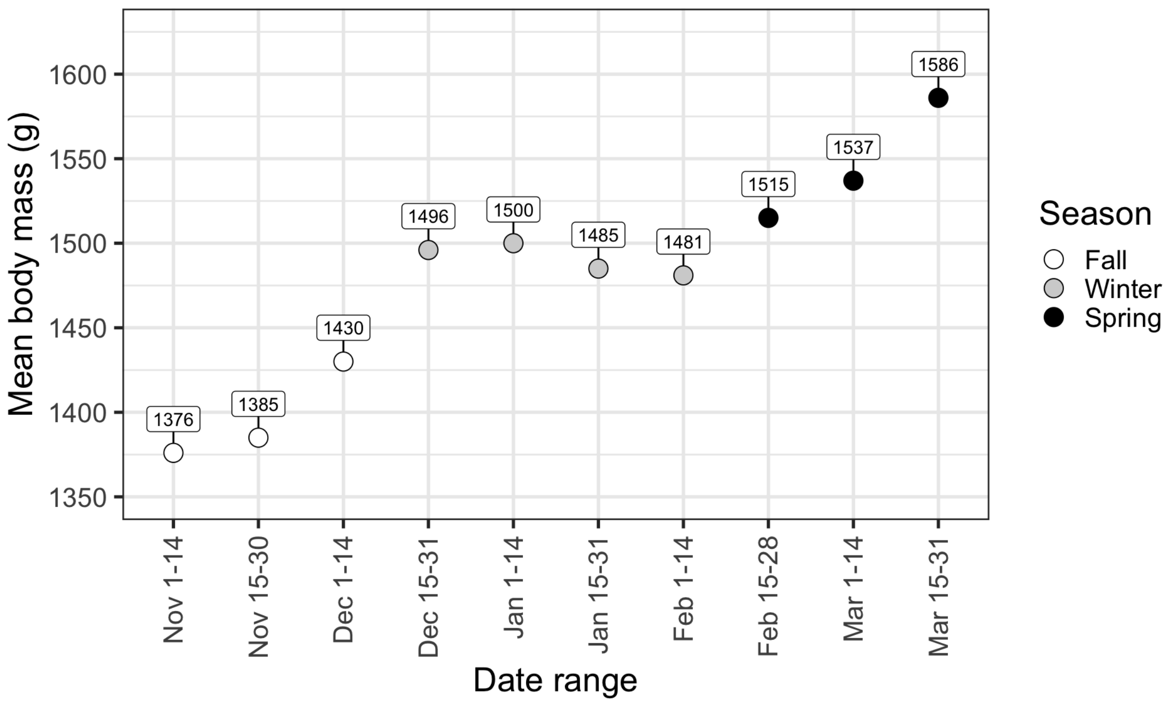

Figure 3. Observed seasonal changes in the body mass of brant in Bahía San Quintín derived from a combination of collected (Mason et al. 2006) and hunted birds (Ward 2024). The symbol shading indicates the dates of the seasons used in the model. Body mass values represent means averaged across age and sex within date ranges (1992–2013).
