# Supplementary material for: Predicting the response of a long‐distance migrant to changing environmental conditions in winter
Source: Ecol Evol. 2024 Jun 29;14(7):e11619. doi: 10.1002/ece3.11619 (PMC11214018; doi:10.1002/ece3.11619)
Supplement: Supplementary file 2 — Appendix S2. [file ECE3-14-e11619-s001.pdf]

APPENDIX S2

Predicting the response of a long-distance migrant to changing environmental conditions in winter

R.A. Stillman[1†], E.M. Rivers[1], W. Gilkerson[2], K.A. Wood[3], P. Clausen[4], C. Deane[5] & D.H. Ward[6]

[1] Department of Life and Environmental Sciences, Faculty of Science and Technology, Bournemouth University, Poole, Dorset, BH12 5BB, United Kingdom.

[2] Merkel & Associates, Inc., 5434 Ruffin Rd, San Diego, CA 92123, United States of America.

[3] Wildfowl & Wetlands Trust, Slimbridge, Gloucestershire, GL2 7BT. United Kingdom.

[4] Department of Ecoscience - Wildlife Ecology, Aarhus University, C.F. Møllers Allé 8, 8000 Aarhus C, Denmark.

[5] Department of Biology and Wildlife, University of Alaska Fairbanks, Fairbanks, Alaska 99775, United States of America.

[6] U.S. Geological Survey, Alaska Science Center, 4210 University Drive, Anchorage, AK 99508-4626, United States of America.

Appendix S2: Tests of the Bahía San Quintín brant model

The model was tested by comparing its predictions to the following observed field data; proportion of birds feeding, staging duration and distribution of birds across subsites. None of these observations were used to parameterise the model. Instead, the equivalent model predictions emerged from the other parameters and processes it contained. For example, comparison of the predicted and observed biomass of eelgrass was not used to test the model, as the seasonal change in eelgrass biomass within the model was calibrated so that predicted biomass matched the observed. As model predictions varied between years, due to between-year variation in biotic and abiotic environmental conditions, observations and predictions were compared for equivalent years. Direct tests of predictions were restricted to years in which observed data were collected.

Proportion of time feeding

The observed time spent feeding by individual birds was not recorded directly, but instead the proportion of flocks feeding was measured from counts throughout the tidal cycle during 2011 and 2012. This was assumed to be comparable with the mean proportion of time spent feeding by individual birds predicted by the model. The mean observed proportion of individuals feeding within flocks during winter and fall was 0.84 in 2011 and 0.79 in 2012, compared to the predicted proportion of time feeding of 0.98 and 0.95 in 2011 and 2012 respectively (Table 1a; Figure 1). This suggests that the model birds were having more difficulty maintaining their energy demands than the real birds, and so needed to spend a longer amount of time feeding to meet these demands. The predicted proportion of time spent feeding varied across other years, in some cases exceeding and in some cases being lower than the observed proportion feeding. 2011 and 2012 were years with relatively low

eelgrass biomass (see main paper), and so it is possible that the birds in the real system exploited some other food resources during these years. This could have reduced the amount of time that the real birds needed to spend feeding on eelgrass as some of their energy demands would have been obtained from other food sources. Eelgrass was the only food resource available to the model birds and so they did not have the option of obtaining some of their energy from other food resources.

Duration of stay

The observed duration of stay was measured from radio-tracked individuals (n = 10 for winter residents and 17 for spring migrants) during 1999 for fall migrants, winter residents and spring migrants (Ward 2024). The duration of stay of fall migrants was not used to test the model, as this was used to parameterise the model; model fall migrants were assumed to stay in the site for the same number of days as the real birds (see main paper). During 1999, the mean observed duration of stay for winter residents was 89.7 days (minimum = 40 days; maximum = 169 days) and for spring migrants was 21.3 days (minimum = 1 day; maximum = 60 days), compared to the predicted durations of stay in this year of 112.0 and 10.0 days for winter residents and spring migrants respectively (Table 1b; Figure 2). For both winter residents and spring migrants, the predicted duration of stay was within the observed range. The duration of stay of birds in the model depended on their arrival date and the date on which they had gained enough energy to depart from the site. Winter residents were predicted to have a longer mean duration of stay than the real birds, suggesting either that they were assumed to be arriving relatively early and/or these birds in the model were gaining mass at a slower rate in spring than the real birds. The predicted mean duration of stay of spring migrants was shorter than the real birds, suggesting either that they were gaining mass at a higher rate

than the real birds or that the real birds were not leaving the site as soon as they gained enough mass to migrate. Furthermore, the observed data were derived from a relatively small number of radio-tracked birds which may not have been representative of the whole population.

Distribution

The observed distribution of birds within the site was measured during 1999 to 2006, and 2011 to 2013 from counts of birds across the three subsites, Bahía Falsa, East Bay and Back Bay (Ward 2024). The model was tested by comparing the predicted and observed proportion of birds within each subsite over these years (Table 1c, d, e; Figure 3,4, 5). The model predicted the observation that the proportion of birds within Back Bay was relatively low compared to the proportions within Bahía Falsa and East Bay. Although the differences between observations and predictions varied between different years, overall, the model predicted a similar proportion of birds to observed within Bahía Falsa and East Bay. The distribution of birds in the model was determined by the distribution of eelgrass biomass and changes in its availability to the birds through the tidal cycle. In the real system, other environmental factors may have influenced the distribution of the birds, for example, the changing abundance of eelgrass during El Nino events affecting distribution of brant in Mexico (Lindberg et al. 2007) and / or the distribution of disturbance from human and natural sources.

References

Lindberg, M. S., Ward, D. H., Tibbitts, T. L., & Roser, J. (2007). Winter movement dynamics of black brant. The Journal of Wildlife Management, 71(2), 534-540.

Ward, D. H., (2024). Data from black brant (Branta bernicla nigricans) overwintering in three lagoons along the Baja California Peninsula, Mexico (ver 2.0, January 2024): U.S. Geological Survey data release, https://doi.org/10.5066/F7T43R88.

Table 1. Observed and predicted (a) proportion of time feeding, (b) duration of stay and (c, d, e) distribution of brant in Bahía San Quintín. Predictions are the mean of five replicate simulations. See Figures 1-5 for details of observations.

(a) Proportion of time feeding

Year	Season	Observed	Predicted
2011	Winter / Spring	0.84 (95% c.i. = 0.05)	0.98
2012	Winter / Spring	0.79 (95% c.i. = 0.06)	0.95

(b) Duration of stay

Year	Bird type	Observed	Predicted
1999	Winter resident	89.7 (95% c.i. = 20.7)	112.0
1999	Spring migrant	21.3 (95% c.i. = 10.9)	10.0

(c) Proportion in Bahía Falsa

Year	Season	Observed	Predicted
1999	Fall	0.34	0.41
2000	Fall	0.36	0.37
2001	Fall	0.17	0.28
2002	Fall	0.35	0.44
2003	Fall	0.2	0.40
2004	Fall	No data	0.39
2005	Fall	No data	0.32
2006	Fall	0.31	0.41
2011	Fall	0.17	0.35
2012	Fall	0.39	0.41
2013	Fall	0.45	0.41
1999	Winter	0.42	0.46
2000	Winter	0.42	0.43
2001	Winter	0.45	0.45
2002	Winter	0.35	0.45
2003	Winter	0.28	0.41
2004	Winter	0.28	0.39
2005	Winter	0.39	0.40
2006	Winter	0.38	0.38
2011	Winter	0.63	0.41
2012	Winter	0.58	0.40
2013	Winter	0.56	0.44
1999	Spring	0.45	0.44
2000	Spring	0.41	0.43
2001	Spring	0.43	0.53
2002	Spring	0.31	0.41
2003	Spring	0.34	0.49
2004	Spring	0.28	0.46
2005	Spring	0.33	0.44
2006	Spring	0.38	0.45
2011	Spring	0.54	0.42
2012	Spring	0.48	0.45
2013	Spring	0.63	0.37

(d) Proportion in East Bay

Year	Season	Observed	Predicted
1999	Fall	0.65	0.59
2000	Fall	0.63	0.61
2001	Fall	0.83	0.68
2002	Fall	0.65	0.56
2003	Fall	0.8	0.57
2004	Fall	No data	0.57
2005	Fall	No data	0.63
2006	Fall	0.69	0.54
2011	Fall	0.8	0.60
2012	Fall	0.37	0.55
2013	Fall	0.36	0.56
1999	Winter	0.55	0.46
2000	Winter	0.52	0.53
2001	Winter	0.54	0.49
2002	Winter	0.65	0.49
2003	Winter	0.72	0.57
2004	Winter	0.72	0.57
2005	Winter	0.59	0.55
2006	Winter	0.62	0.59
2011	Winter	0.34	0.55
2012	Winter	0.28	0.57
2013	Winter	0.37	0.51
1999	Spring	0.49	0.52
2000	Spring	0.55	0.53
2001	Spring	0.54	0.44
2002	Spring	0.68	0.54
2003	Spring	0.66	0.48
2004	Spring	0.72	0.52
2005	Spring	0.67	0.52
2006	Spring	0.61	0.54
2011	Spring	0.36	0.55
2012	Spring	0.44	0.53
2013	Spring	0.36	0.60

(e) Proportion in North Bay

Year	Season	Observed	Predicted
1999	Fall	0.01	0.00
2000	Fall	0.01	0.02
2001	Fall	0	0.04
2002	Fall	0	0.00
2003	Fall	0	0.03
2004	Fall	No data	0.04
2005	Fall	No data	0.05
2006	Fall	0	0.05
2011	Fall	0.02	0.06
2012	Fall	0.24	0.04
2013	Fall	0.18	0.03
1999	Winter	0.04	0.08
2000	Winter	0.06	0.05
2001	Winter	0.01	0.06
2002	Winter	0	0.07
2003	Winter	0	0.02
2004	Winter	0	0.04
2005	Winter	0.02	0.05
2006	Winter	0	0.03
2011	Winter	0.03	0.04
2012	Winter	0.14	0.04
2013	Winter	0.07	0.05
1999	Spring	0.06	0.03
2000	Spring	0.04	0.04
2001	Spring	0.03	0.03
2002	Spring	0.01	0.05
2003	Spring	0	0.03
2004	Spring	0	0.02
2005	Spring	0	0.04
2006	Spring	0.01	0.02
2011	Spring	0.1	0.03
2012	Spring	0.08	0.01
2013	Spring	0.01	0.03

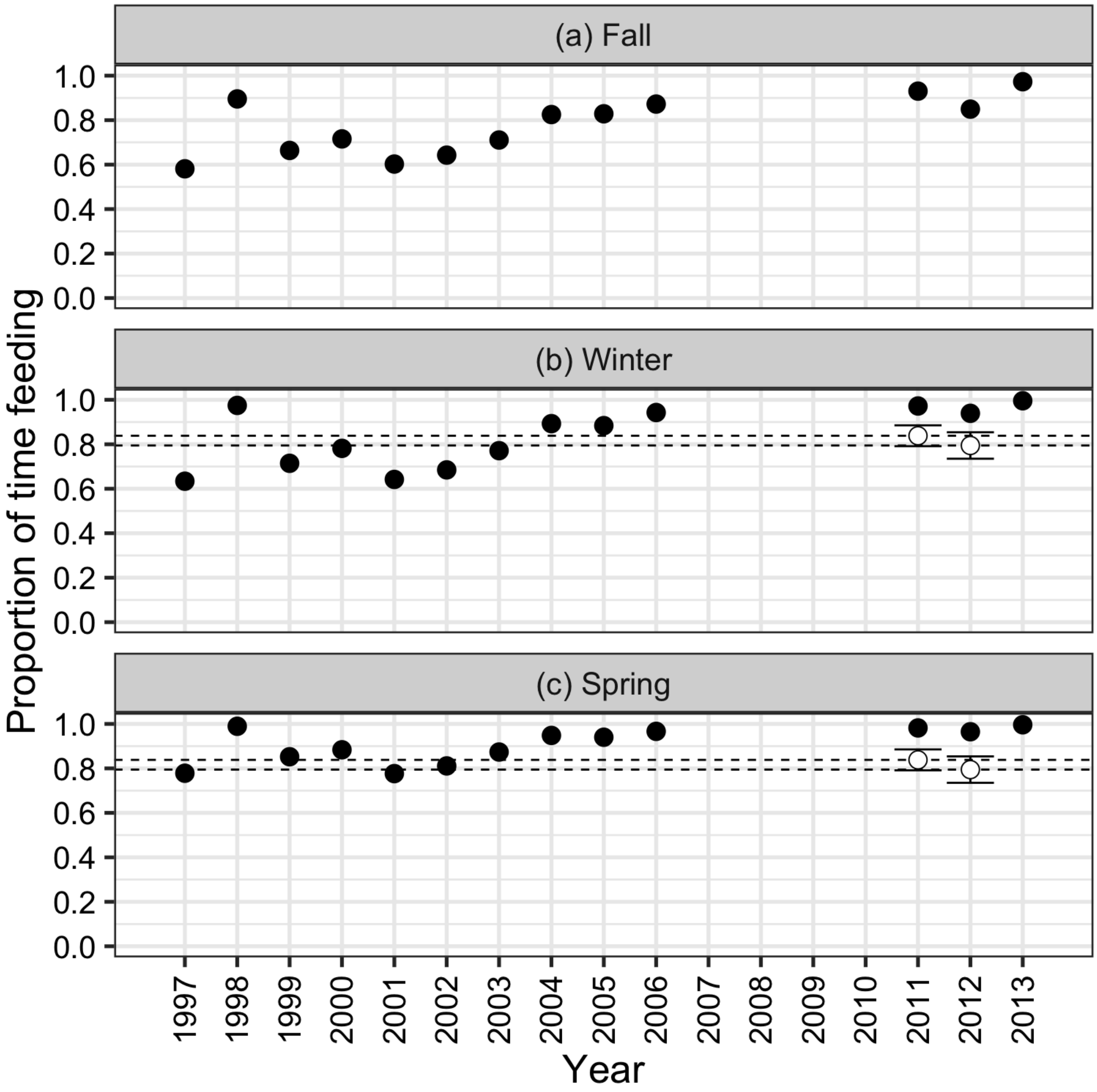

Figure 1. Predicted (solid symbols) and observed (open symbols, dashed lines) proportion of time spent feeding by brant during daylight in Bahía San Quintín. Observations are from counts of the number of individuals with different behaviours in flocks made during January to March in 2011 and 2012. It is assumed that the mean observed proportion of birds feeding equates to the mean predicted proportion of time spent feeding. Data from these months is included for both winter and spring. Observed symbols show mean and 95% confidence intervals of proportion of birds feeding (weighted by flock size, excluding flying birds). Horizontal dashed lines indicate how predictions across all years compare to the years from which observations were made. Predictions are the mean of five replicate simulations.

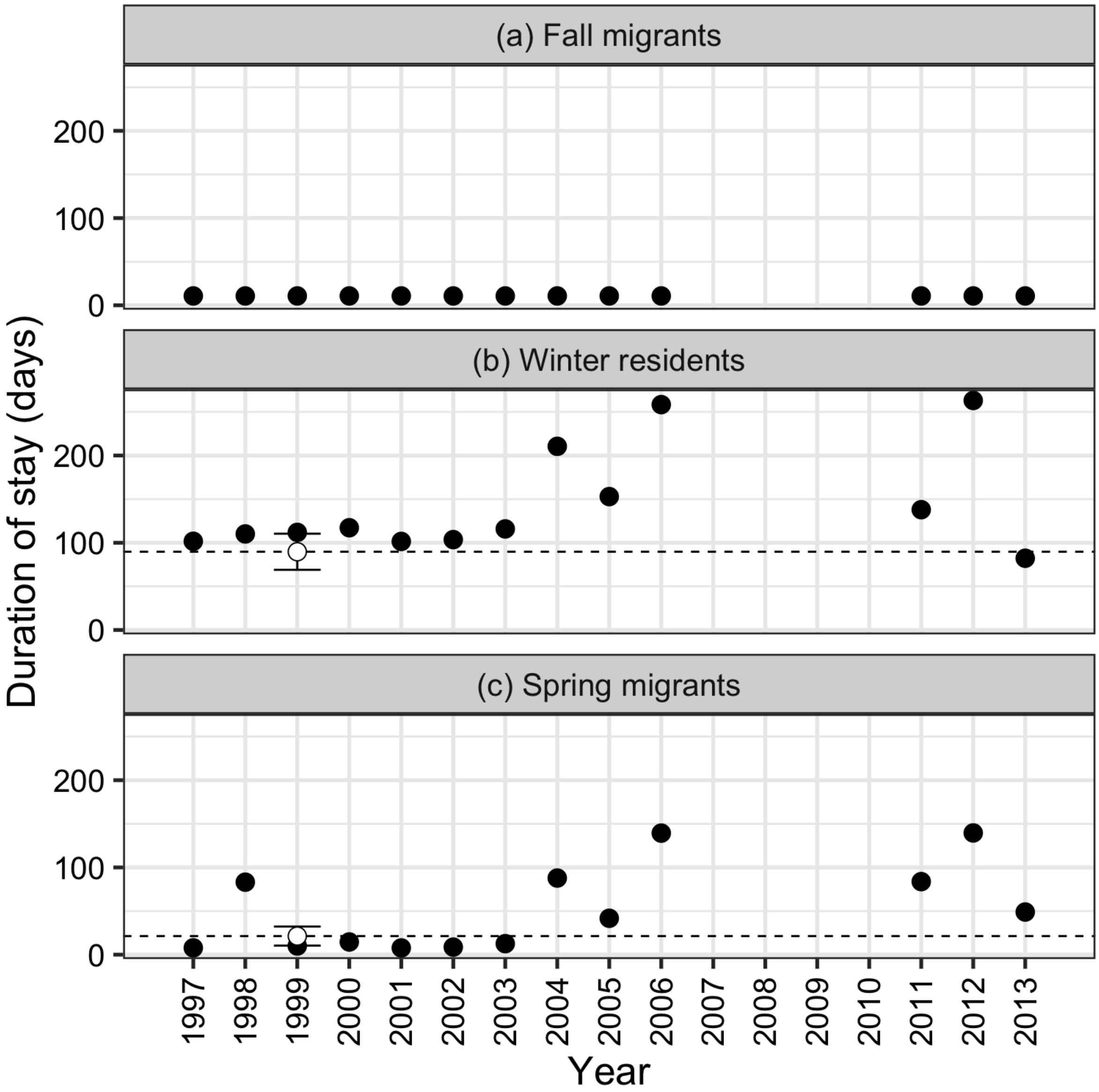

Figure 2. Predicted (solid symbols) and observed (open symbols, dashed lines) duration of stay of brant at Bahía San Quintín. Observations are from radio tracked individuals during 1999. Observed symbols show mean and 95% confidence intervals of duration of stay. Horizontal dashed lines indicate how predictions across all years compare to the years from which observations were made. Predictions are the mean of five replicate simulations.

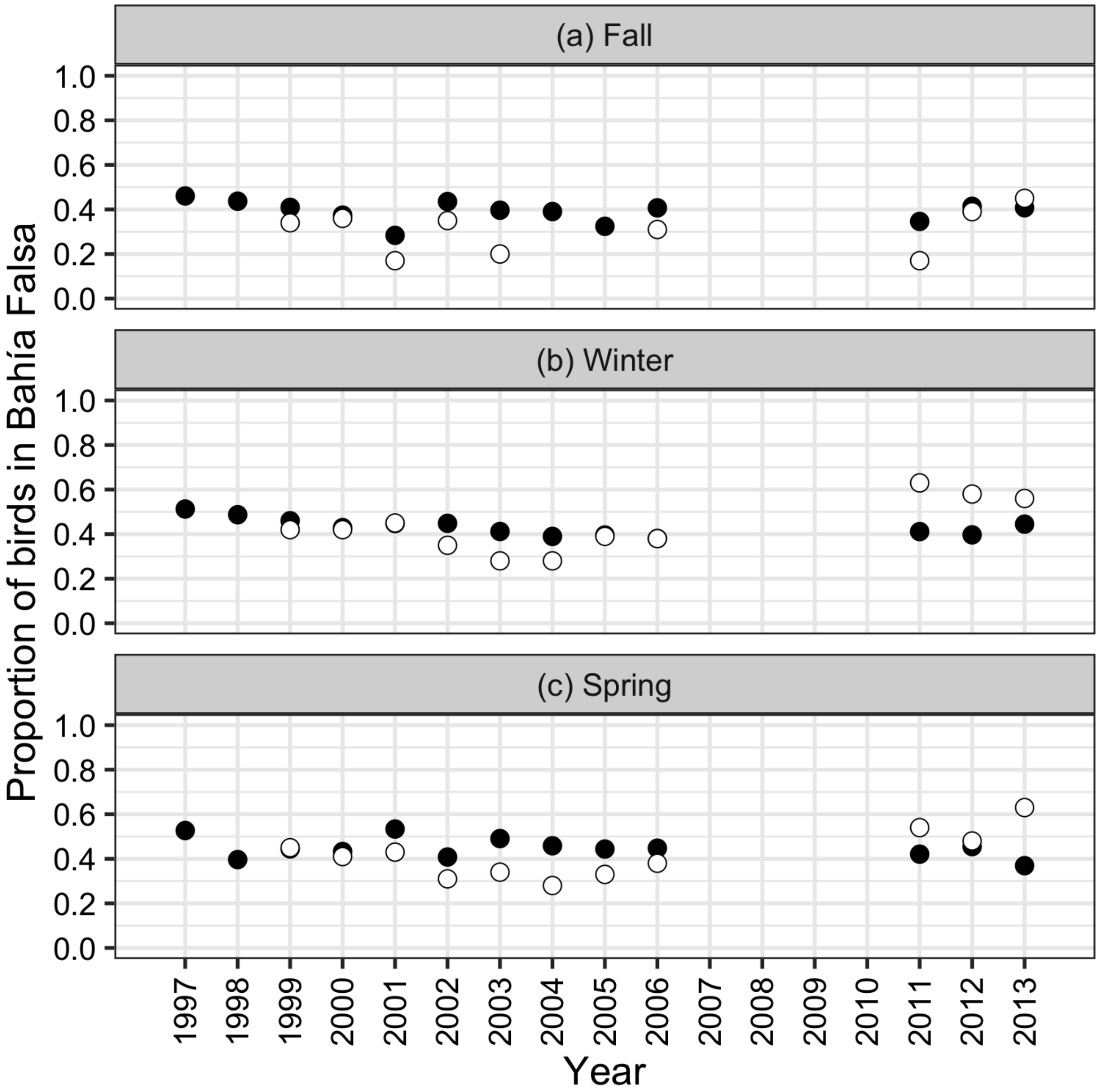

Figure 3. Predicted (solid symbols) and observed (open symbols) proportion of birds within Bahía Falsa at Bahía San Quintín. Observations are from counts made during 1999 to 2006, and 2011 to 2013 (no data were collected during fall 2004 and 2005; Ward 2024). Predictions are the mean of five replicate simulations.

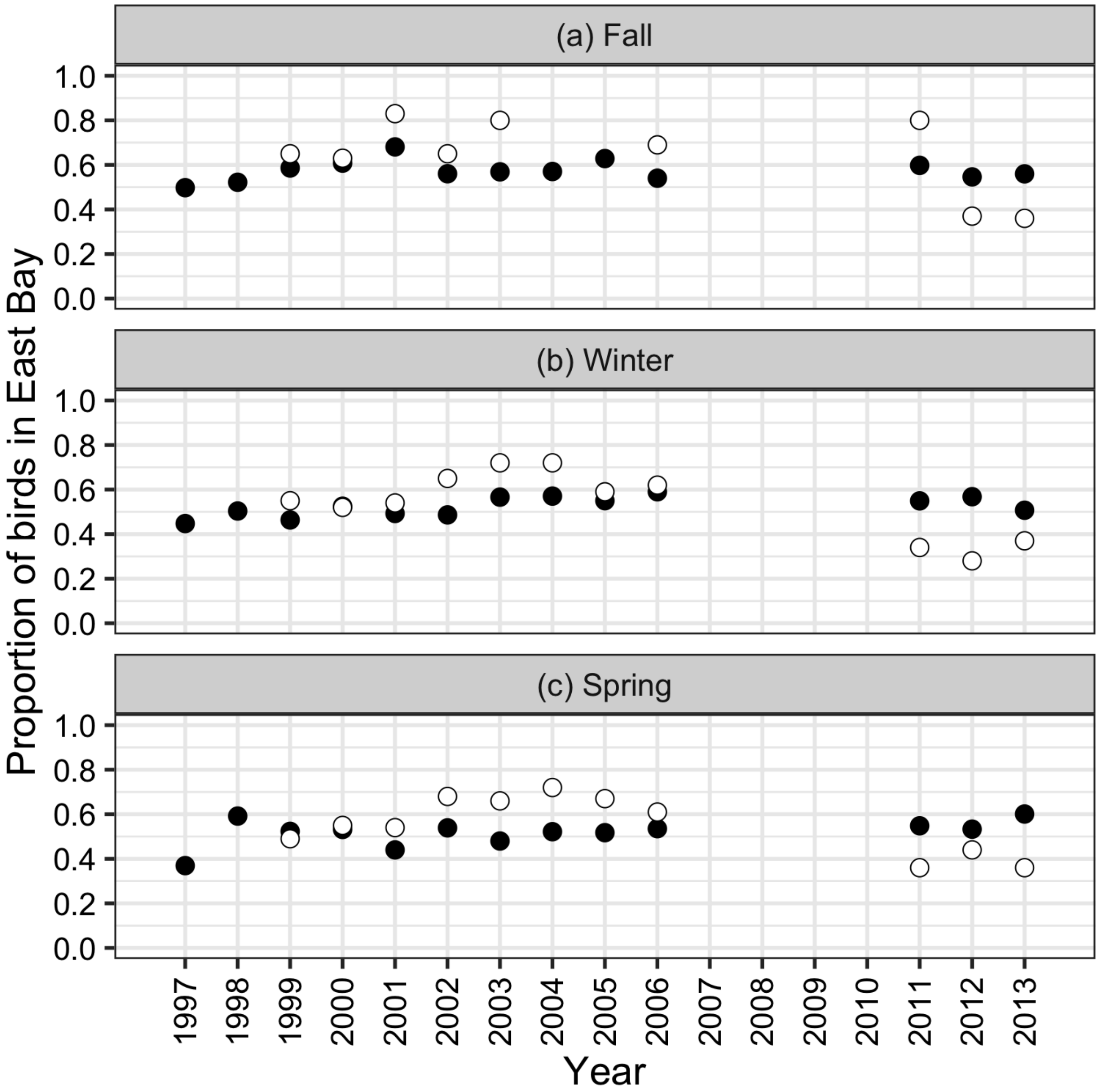

Figure 4. Predicted (solid symbols) and observed (open symbols) proportion of birds within East Bay at Bahía San Quintín. Observations are from counts made during 1999 to 2006, and 2011 to 2013 (no data were collected during fall 2004 and 2005; Ward 2024). Predictions are the mean of five replicate simulations.

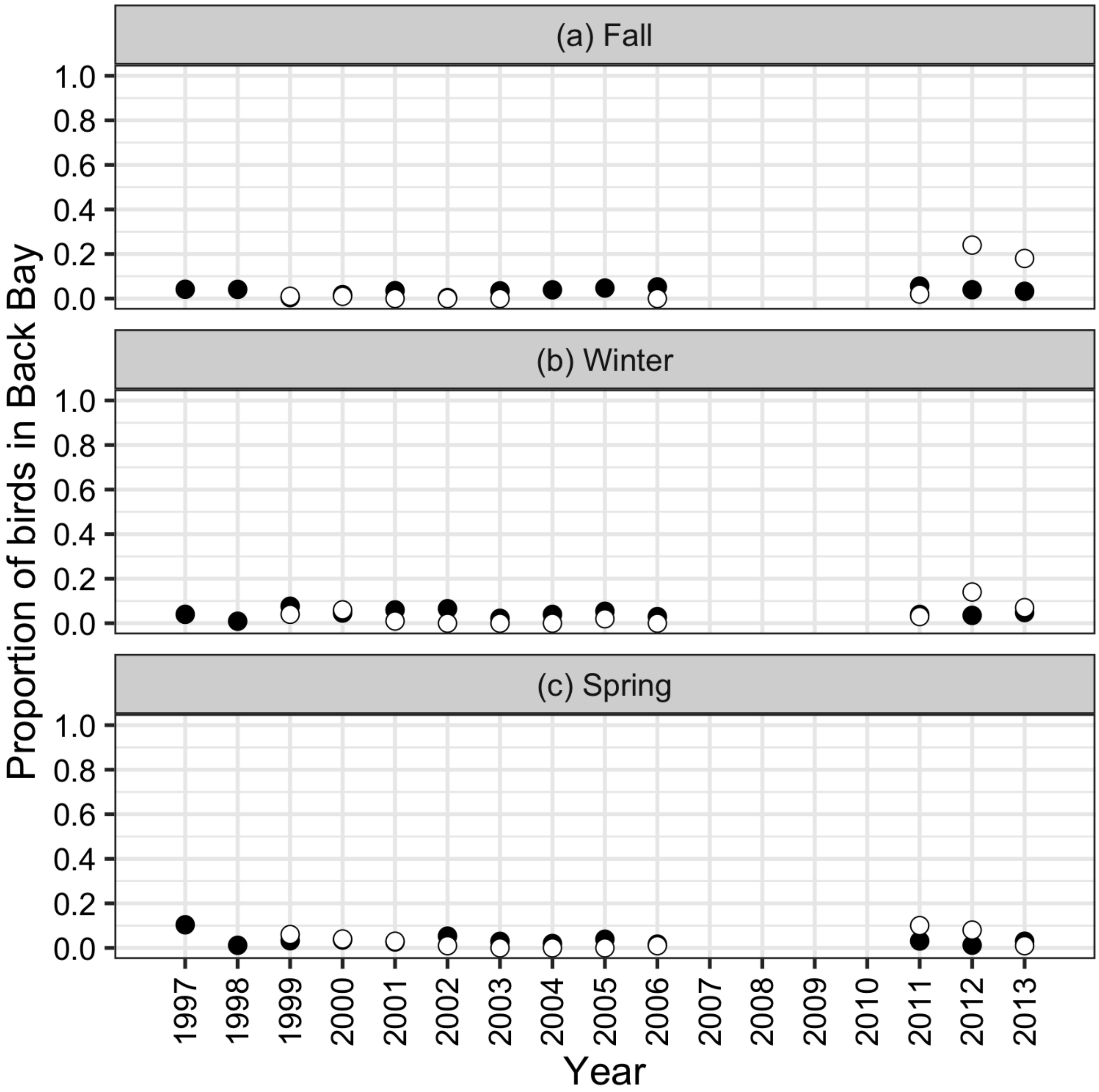

Figure 5. Predicted (solid symbols) and observed (open symbols) proportion of birds within Back at Bahía San Quintín. Observations are from counts made during 1999 to 2006, and 2011 to 2013 (no data were collected during fall 2004 and 2005; Ward 2024). Predictions are the mean of five replicate simulations.
